# The HIF-1α as a Potent Inducer of the Hallmarks in Gastric Cancer

**DOI:** 10.3390/cancers14112711

**Published:** 2022-05-30

**Authors:** Cemre Ucaryilmaz Metin, Gulnihal Ozcan

**Affiliations:** 1 Graduate School of Health Sciences, Koç University, 34450 Istanbul, Turkey; cucaryilmaz20@ku.edu.tr; 2 Department of Medical Pharmacology, School of Medicine, Koç University, 34450 Istanbul, Turkey

**Keywords:** gastric cancer, hypoxia, hypoxia-inducible factor-1α, hallmarks of cancer

## Abstract

**Simple Summary:**

Gastric cancer is one of the most aggressive tumors in the clinic that is resistant to chemotherapy. Gastric tumors are rich in hypoxic niches, and high expression of hypoxia-inducible factor-1α is associated with poor prognosis. Therefore, strategies that target hypoxia-inducible factor-1α signaling may be highly effective in gastric cancer treatment. However, the precise mechanisms by which hypoxia-inducible factor-1α induces tumor hallmarks in gastric cancer are yet unrevealed. Here, we review the role of hypoxia-inducible factor-1α as a potent inducer of the cancer hallmarks in gastric cancer to provide a broad perspective and reveal missing links investigating which may offer new strategies to target hypoxia-inducible factor-1α signaling in gastric cancer.

**Abstract:**

Hypoxia is the principal architect of the topographic heterogeneity in tumors. Hypoxia-inducible factor-1α (HIF-1α) reinforces all hallmarks of cancer and donates cancer cells with more aggressive characteristics at hypoxic niches. HIF-1α potently induces sustained growth factor signaling, angiogenesis, epithelial–mesenchymal transition, and replicative immortality. Hypoxia leads to the selection of cancer cells that evade growth suppressors or apoptotic triggers and deregulates cellular energetics. HIF-1α is also associated with genetic instability, tumor-promoting inflammation, and escape from immunity. Therefore, HIF-1α may be an important therapeutic target in cancer. Despite that, the drug market lacks safe and efficacious anti-HIF-1α molecules, raising the quest for fully unveiling the complex interactome of HIF-1α in cancer to discover more effective strategies. The knowledge gap is even wider in gastric cancer, where the number of studies on hypoxia is relatively low compared to other well-dissected cancers. A comprehensive review of the molecular mechanisms by which HIF-1α induces gastric cancer hallmarks could provide a broad perspective to the investigators and reveal missing links to explore in future studies. Thus, here we review the impact of HIF-1α on the cancer hallmarks with a specific focus on gastric cancer.

## 1. Introduction

Gastric cancer (GC) ranks the fifth most common cancer worldwide [1]. Localized GC is commonly treated with gastrectomy and lymphadenectomy plus chemotherapy/chemoradiotherapy. However, the disease is mostly diagnosed at the metastatic stage. In advanced-stage inoperable GC patients, combination chemotherapy is the mainstay of treatment [2,3]. Conventional chemotherapeutics with different mechanisms of action, such as fluoropyrimidines, platinum coordination complexes, and taxanes, take part in the combination protocols [4,5]. However, unfortunately, resistance to conventional chemotherapeutics is a big handicap [6].

With advancements in targeting tumor-specific processes, molecular-targeted agents and immunotherapeutics are incorporated into the GC treatment protocols. Ramucirumab (anti-VEGFR2), trastuzumab (anti-HER2), nivolumab (anti-PD-1), and pembrolizumab (anti-PD-1) are approved for use in GC treatment protocols. These agents improved treatment outcomes [3,4,7,8,9,10,11,12,13,14], and overall survival rates of GC patients are increasing throughout the world [15]. Clinical trials are being conducted to translate new molecular-targeted agents into the GC treatment that target claudin-18.2 (NCT03505320, NCT04400383, NCT05009966) and FGF2 (NCT05052801, NCT05322577, NCT05019794). However, molecular-targeted agents and immunotherapy are indicated in advanced-stage patients with target positivity, which excludes a large group of GC patients [16], and GC is still the third leading cause of cancer-related deaths [1]. Furthermore, projections point out an increase in the burden of gastric cancer in many countries, especially in Eastern Asia [17]. Thus, new molecular-targeted agents that would be effective in a larger group of GC patients are required.

The tumor microenvironment bears many opportunities for discovering new molecular targets in GC. Dynamic interaction of the cancer cells with the cellular and non-cellular compartments of its microenvironment sustains the adaptive evolution of the tumors, which guarantees the survival of cancer cells despite the presence of cytotoxic chemotherapeutics [18]. The cellular components of the tumor microenvironment such as tumor-infiltrating lymphocytes, tumor-associated macrophages, and cancer-associated fibroblasts, and non-cellular components such as ECM proteins, inflammatory mediators, and growth factors fertilize the soil for tumor progression and chemoresistance in GC [19,20].

Biophysical factors play a pivotal role in architecting both the cellular and non-cellular compartments of the tumor microenvironment. Insufficient or abnormal vascularity induces gradients of oxygen, pH, nutrients, growth factors, cytokines, interstitial pressure, and blood-borne chemotherapeutics within the tumor mass. The heterogeneous distribution of these factors brings distinct characteristics to the tumor cells at different niches in terms of proliferation rate, capabilities for invasion and metastasis, and propensity for destruction by immune cells or chemotherapeutics [18,21,22].

Hypoxia is an indispensable prognostic factor in the microenvironment of gastric tumors. Gastric tumors embody widespread hypoxic niches [23,24]. Nuclear magnetic resonance spectroscopy studies revealed that the residence of severe or moderate hypoxia in the primary tumor is associated with an increased frequency of disseminated tumor cells in the bone marrow [25] and poor overall survival in GC patients. Hypoxia was associated with a more than two-fold increase in mortality, even in patients with no lymph node involvement [26]. Hypoxia was also observed in the normal gastric mucosa of 52% of the GC patients. The overall survival was significantly worse in these patients [27]. Therefore, a comprehensive understanding of the impact of hypoxia on GC is essential to developing new strategies that can increase patient survival.

Tumor cells at hypoxic niches operate different strategies to adapt to hypoxia, such as favoring glycolytic metabolism over oxidative phosphorylation, increasing genomic instability, developing resistance to apoptosis, acquiring the ability for unlimited proliferation, developing new mechanisms to escape the immune attack, and migrating to less hypoxic areas [28,29,30]. Hypoxia-inducible factor 1α (HIF-1α) plays a pivotal role in these adaptation mechanisms leading to tumor progression and chemoresistance [31,32].

**Figure 1:**
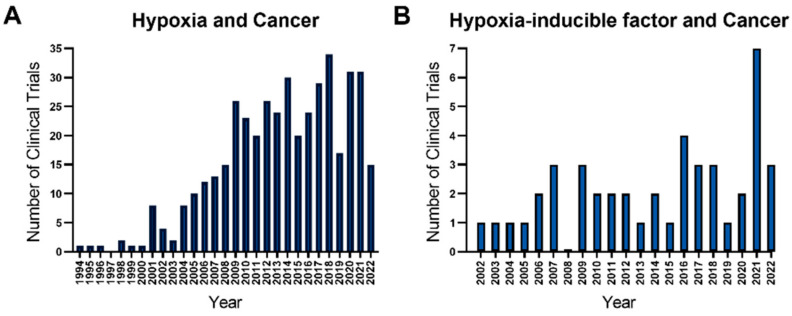
The number of clinical studies in cancer registered on https://clinicaltrials.gov that mention the impact of hypoxia (**A**), and hypoxia-inducible factor (**B**). We searched https://clinicaltrials.gov/ct2/home (accessed on 24 May 2022) to find all the clinical trials registered till 24 May 2022 that mention the terms “hypoxia” or “hypoxia-inducible factor”. The number of studies started each year was counted from the compiled list to draw the graphs in the figure with GraphPad Prism 9. The counts for 2022 only represent the studies started between 1 January 2022 and 24 May 2022.

The extent of information about the molecular mechanisms by which HIF-1α leads to hypoxic adaptation in cancer is increasing at a great pace. In parallel, the awareness of the importance of hypoxia and HIF-1α in cancer therapy is expanding. The number of clinical trials in cancer that mention “hypoxia” or “hypoxia-inducible factor” increased over the years (Figure 1A,B) (https://clinicaltrials.gov/ct2/home, accessed on 24 May 2022). Numerous drugs that inhibit the transcription, translation, stability, dimerization, or DNA binding of HIF-1α are being tested in clinical trials [33]. In addition to the endeavors to translate HIF-1α antisense oligonucleotides into cancer therapy [34,35,36], several approved chemotherapeutics such as camptothecins, rapamycin analogs, and anthracyclines have been tested clinically for their indirect HIF-1α inhibitory action in various cancers [37]. Nevertheless, the failure rate is high with HIF-1α inhibitors [38], which points out the need to delineate precise molecular mechanisms by which HIF-1α impacts cancer cells. The knowledge gap is even wider in GC, one of the most aggressive cancers in the clinic. Accordingly, there is only one registered trial on https://clinicaltrials.gov testing the impact of inhibiting HIF-1α on the treatment outcome in GC to the best of our knowledge (NCT01049620). In the corresponding study, the RAD001 that inhibits PI3K/AKT and HIF-1α has been tested for its synergistic effect with capecitabine and oxaliplatin in advanced-stage GC patients.

Uncovering the precise mechanisms by which HIF-1α induces tumor progression and chemoresistance in GC may offer new strategies to target the HIF-1α pathway and improve treatment outcomes in GC. Basic and translational studies in this direction may increase the number of clinical trials in GC that will test the impact of direct or indirect inhibitors of HIF-1α, in the following years. Hence in this review, we aim to provide a comprehensive insight into the molecular mechanisms by which HIF-1α induces GC progression and the missing links to be dissected in future studies.

For the review, we searched PubMed for the research articles on hypoxia signaling in cancer with a specific focus on GC. We used the PubMed MeSH terms “hypoxia and stomach neoplasms”, “HIF-1α and stomach neoplasms”, “hypoxia and neoplasms”, and “HIF-1α and neoplasms” to search the literature for studies published till 1 January 2022. Then, we sub-classified the articles for the terms “HIF-1α regulators in cancer”, “HIF-1α and sustained growth factor signaling”, “HIF-1α and evasion from growth suppressors”, “HIF-1α and resistance to apoptosis”, “HIF-1α and replicative immortality”, “HIF-1α and angiogenesis”, “HIF-1α and epithelial–mesenchymal transition”, “HIF-1α and genetic instability”, “HIF-1α and deregulation of cellular energetics”, “HIF-1α and escape from immunosurveillance”, and “HIF-1α and tumor-promoting inflammation”. In each section and subsection, we first summarized the role of hypoxia/HIF-1α on hallmarks of cancer as a background, then we reviewed the specific literature on GC. In the network diagrams in the following sections, we presented the processes/signaling molecules that commonly take part in HIF-1α signaling in diverse cancer types with pink, and processes/signaling molecules for which there is specific evidence in GC with blue.

## 2. HIF-1α and Gastric Cancer

**Figure 2:**
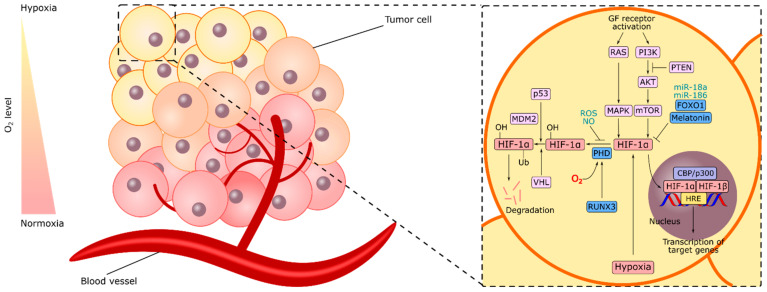
Regulation of HIF-1α in hypoxic cancer cells. Common regulators in different cancers are shown in light pink. Regulators for which there is specific evidence in gastric cancer are shown in blue.

Hypoxia-inducible factors (HIFs) are the central elements for adaptation to hypoxia both in healthy tissues and tumors [39]. These heterodimeric transcription factors consist of HIF-α and HIF-β subunits. HIF-α has three isoforms, HIF-1α, HIF-2α, and HIF-3α. Under normoxic conditions, prolyl hydroxylases (PHDs) hydroxylate HIF-α in a reaction where O_2_ is required (Figure 2). The hydroxylated HIF-α is ubiquitinated and targeted for proteasomal degradation by a process where von Hippel–Lindau (pVHL) tumor suppressor protein is involved. In hypoxic conditions, HIF-α becomes stable due to the lack of oxygen, translocates to the nucleus, and couples with HIF-β, constitutively expressed independently from the oxygen pressure in the environment [40]. Then, the HIF-α and HIF-β complex binds to the hypoxia-response elements, stimulating the expression of genes associated with angiogenesis, glucose metabolism, cell proliferation, and survival, promoting adaptation to hypoxia.

HIF-1, a heterodimer of HIF-1α and HIF-1β, plays the most prominent role in oxygen homeostasis and cancer progression [29]. HIF-1α expression is significantly correlated with aggressive tumor phenotype and poor prognosis in GC [41]. A meta-analysis of nine studies with 1103 subjects demonstrated that half of the GC patients have HIF-1α-expressing tumors. HIF-1α expression was associated with lower 5-year survival, increased depth of invasion, higher risk of lymphatic/vascular invasion, and advanced TNM stage [42]. HIF-1α expression was prominent at the invasive margins and necrotic areas in immunohistochemical staining of GC specimens [43,44,45]. The risk of lymph node metastasis, peritoneal dissemination, and liver metastasis was significantly higher in GC patients with increased expression of HIF-1α [23,24,46].

HIF-1α not only features as a factor that leads to tumor progression but is also a possible mediator in gastric carcinogenesis. HIF-1α expression increases in parallel to the progression from *H. pylori*-associated gastritis to intestinal metaplasia, dysplasia, and intestinal-type GC [45]. The frequency of HIF-1α positivity was even higher in diffuse-type GC samples, including signet ring cell carcinoma and poorly differentiated adenocarcinoma, compared to intestinal-type GC resection samples in two different studies [45,47]. These suggest that the impact of HIF-1α may be more prominent in diffuse-type GC, which warrants the delineation of underlying mechanisms.

Other than hypoxia, several factors regulate HIF-1 activity (Figure 2). For instance, ROS and nitric oxide (NO) can increase HIF-1 activity via the stabilization of HIF-1α. Overactivity of the RAS/MAPK and PI3K/AKT/mTOR oncogenic signaling pathways potentiate HIF-1α activity [48]. Tumor suppressor proteins pVHL, phosphatase and tensin homolog (PTEN), and p53 act like negative regulators of HIF-1α. PTEN negatively regulates the activity of the PI3K/AKT/mTOR pathway, suppressing the induction of HIF-1α expression [41,49]. On the other hand, p53 induces ubiquitination of HIF-1α via MDM2 and leads to proteasomal degradation in specific cancers [50].

Studies in GC suggested PHD2 and PHD3 as the negative regulators of HIF-1 [51,52]. In a cohort of 121 gastric carcinoma patients, PHD2 was positive in 52.9% of the tumor samples. PHD2 negativity was associated with decreased survival in the cohort [51]. PHD3 was shown to be downregulated in gastric tumors compared with the adjacent normal gastric tissue. PHD3 negativity was associated with poorly differentiated phenotype, advanced stage, and lymph node metastasis. Overexpression of PHD3 in AGS GC cells decreased the expression of HIF-1α, and silencing PHD3 in MKN28 GC cells increased the expression of HIF-1α [52]. RUNX family transcription factor 3 (RUNX3), a transcription factor that negatively regulates HIF-1α in GC, increased the binding of HIF-1α to PHD2 and enhanced its degradation in GC cells. Thus, RUNX3 exhibited tumor suppressor action [53]. The downregulation of RUNX3 was associated with poor prognosis in GC [54]. These findings suggest that increasing the activity of PHD2, PHD3, or RUNX3 may be a potential strategy to inhibit HIF-1α in cancer treatment.

Several studies put forth other negative regulators of HIF-1α, downregulation of which may have crucial roles in gastric carcinogenesis and cancer progression (Figure 2) such as FOXO1, melatonin, and specific miRNAs. FOXO1 is the mammalian forkhead transcription factor of the O class 1. It is dysregulated in GC. Under hypoxic conditions, silencing of FOXO1 increased HIF-1α expression in GC cells and xenograft models accompanied by increased angiogenesis and tumor growth [55]. In SGC-7901 GC cells, melatonin decreased the stability and expression of HIF-1α through inhibition of melatonin nuclear receptor RZR/RORγ under hypoxic conditions [56]. Moreover, microRNAs miR-18a and miR-186 target HIF-1α directly and act as negative regulators of HIF-1α in GC [57,58]. It may be straightforward to operate the melatonin and mimics of the miR-18a or miR-186 as an approach to inhibit HIF-1α signaling in GC. Thus, the field may obtain extensive benefit from the investigation of their negative role with further details.

## 3. HIF-1α and Hallmarks in Gastric Cancer

### 3.1. HIF-1α and Sustained Growth Factor Signaling in Gastric Cancer

**Figure 3:**
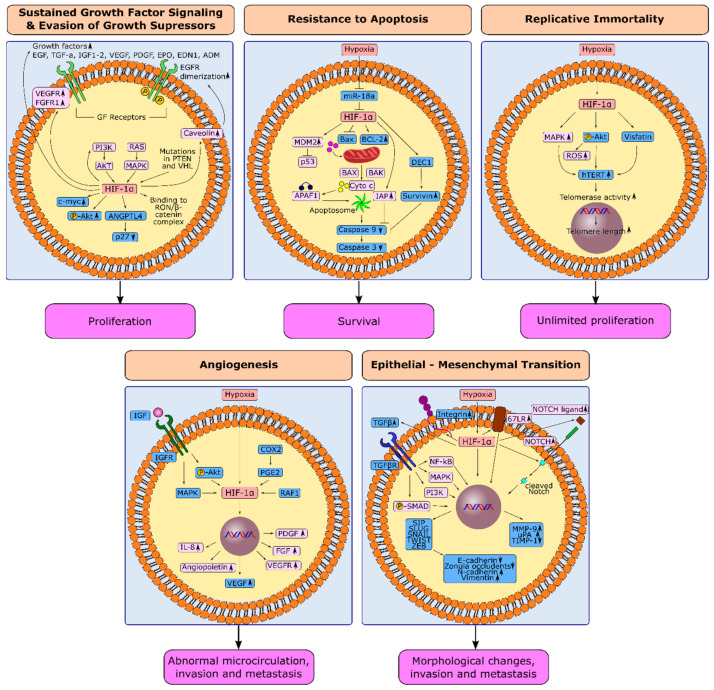
Induction of cancer hallmarks by hypoxia and HIF-1α in gastric cancer. Hypoxia induces sustained growth factor signaling and evasion from growth suppressors, resistance to apoptosis, replicative immortality, angiogenesis, and epithelial–mesenchymal transition in gastric cancer. Processes for which there is specific evidence in gastric cancer are shown in blue. Processes common in different cancers are shown in light pink. Upregulation or downregulation of specific proteins is shown with an upward or downward arrow, respectively. The figures were drawn in Inkscape 1.1.2.

Growth factor (GF) signaling is tightly regulated in normal tissues to control the cell number and maintain homeostasis. Cancer cells circumvent this regulation by producing GFs themselves, upregulating the GF receptors (GFRs), or employing constitutive active signaling proteins downstream to the GF receptors. This way, dependence on GFs and GFRs for cellular growth is eliminated [59]. Hypoxia potently induces these mechanisms leading to the sustained proliferation of cancer cells (Figure 3).

In a hypoxic microenvironment, HIFs can induce the synthesis of various GFs, including epidermal growth factor (EGF), transforming growth factor-α (TGF- α), insulin-like growth factors 1 and 2 (IGF1 and IGF2), platelet-derived growth factor (PDGF), endothelin1 (EDN1), adrenomedullin (ADM) and erythropoietin (EPO) in renal cell carcinoma, colorectal carcinoma, pancreatic cancer, breast cancer, prostate cancer, melanoma, and ovarian cancer cells [60,61]. Among the GFRs, hypoxia increased the expression of fibroblast growth factor receptor 1 (FGFR1) via the MAPK signaling pathway in lung cancer cell lines and xenograft models [62]. Both vascular endothelial growth factor (VEGF) and VEGF receptor 1 (VEGFR1) are upregulated by HIF-1α and boost the proliferation of endothelial cells leading to angiogenesis, as shown in endothelial cells isolated from the human umbilical vein and pig aorta [63]. Moreover, GFRs can be constitutively active in a hypoxic environment and stimulate cell proliferation regardless of the presence of GFs. For instance, HIF-induced upregulation of the caveolin increased EGFR dimerization and phosphorylation, leading to EGF-independent activation of EGFR signaling in renal cell carcinoma cell lines [64]. In addition to the regulatory role of HIF-1α on GF signaling pathways, downstream signaling molecules in GF pathways also seem to have control over HIF-1α, suggesting the possibility of a positive feedback interaction between these two. The overactivity of RAS/MAPK and PI3K/AKT/mTOR pathways upregulates HIF-1α [41]. Findings in vulvar squamous adenocarcinoma, colorectal carcinoma, and non-small cell lung cancer cell lines suggest that EGFR can induce HIF-1α expression by the PI3K/AKT pathway [65].

In GC, it requires a laborious effort to find studies that demonstrate a direct relationship between hypoxia and increased expression of GFs or GFRs. Except for VEGFR. On the other hand, a reasonable number of studies in GC suggest that hypoxia regulates GF signaling through the activation of signaling mechanisms downstream of the GFRs (Figure 3). Recepteur d’origine nantais (RON) is a member of the c-met family of receptor tyrosine kinases that induce cell proliferation, migration, and invasion via activating oncogenic signaling pathways. RON was reported to be upregulated in GC tissues. It is not clear whether hypoxia controls RON expression. Nonetheless, it was observed that binding of HIF-1α to the RON/β-catenin complex increases under hypoxic conditions and enhances the proliferation of GC cell lines [66].

In GC in vitro models, HIF-1α acted as an upstream regulator of AKT phosphorylation, inducing cell proliferation [67]. Hypoxia increased c-Myc oncogene expression and decreased cell-cycle inhibitor p27 expression through “angiopoietin like-4” (ANGPTL4) protein, and stimulated cell proliferation in scirrhous GC cell models, which is a highly aggressive and metastatic type of GC [68]. In GC patients, HIF-1α expression correlated with the phosphorylation of AKT. In GC cell lines that exhibit constitutive activation of AKT, an increase in HIF-1α expression was reported even in normoxic conditions [69]. Despite that, the GFRs that might be upregulated or constitutively activated by HIF-1α are unclear in GC. Identifying these GFRs may unlock the opportunity to target HIF-1α-induced oncogenic pathways in GC, via targeting corresponding GFRs. This possibility may enable the combination of GFR inhibitors with HIF-1α inhibitors in a synergistic manner.

### 3.2. HIF-1α and Evasion from Growth Suppressors in Gastric Cancer

Cancer cells breach the suppressive actions of tumor suppressor genes on cellular growth through genetic and epigenetic alterations [59,70]. Decreased expression of tumor suppressors PTEN and pVHL is associated with poor prognosis in GC [71,72,73]. The mutations in tumor suppressor genes PTEN and pVHL are associated with the upregulation of HIF-1α which may contribute to poor prognosis in PTEN or pVHL-deficient cancers [41,49] (Figure 3). Hypoxia may also pose a selective pressure leading to the selection of GC cells with dysfunctional tumor suppressor genes. Notably, concomitant overexpression of tumor suppressor protein p53 with HIF-1α was correlated with a dismal prognosis in GC patients, where HIF-1α(+)/p53(+) primary gastric tumors were more frequently associated with an undifferentiated, infiltrative, and metastatic phenotype compared to HIF-1α(−)/p53(−) tumors [74]. The authors did not investigate whether p53(+) tumor samples expressed wild-type or mutant p53 in the study. However, extensive literature suggests that selective pressure posed by hypoxia may lead to enrichment of cells with loss of function mutations in p53 and could explain the dismal prognosis in HIF-1α(+)/p53(+) tumors [75]. This may explain the failure of HIF-1α inhibitors since selected cancer cells will be inherently resistant to apoptosis in these circumstances. Therefore, p53 expression and mutation status of hypoxic cells in gastric tumors should be elucidated as a prospect for the possible use of HIF-1α inhibitors in GC.

### 3.3. HIF-1α and Resistance to Apoptosis in Gastric Cancer

The evasion of cancer cells from apoptotic triggers is a hallmark of cancer that is crucial for tumor progression and resistance to cancer therapy [59]. Hypoxia triggers apoptosis through a HIF-1α-mediated increase in mitochondrial membrane permeability. As a result, cytochrome c is released from the mitochondria and induces p53-dependent apoptosis where APAF1 and caspases are involved [29]. Additionally, HIF-1α induces caspase-independent cell death through upregulation of BNIP3 and NIX, pro-apoptotic members of the BCL-2 family [76]. Activated JNK is also involved in hypoxia-induced apoptosis [77].

Although hypoxia itself is an apoptotic trigger, cancer cells that can escape from the apoptotic action of hypoxia are selected under hypoxic pressure, generating tumors with a more aggressive and resistant clonal composition [29,78]. Under recurrent hypoxia, p53-mutant and apoptosis-deficient subpopulations are selected remarkably and expand rapidly [78]. Hypoxia was shown to induce anti-apoptotic mechanisms simultaneously with pro-apoptotic processes in ovarian cancer cell lines. Hypoxia conferred resistance to apoptosis by promoting the expression of anti-apoptotic protein BCL-2 in ovarian cancer cell lines, apoptosis inhibitory protein IAP-2 in proximal tubule cells from rat kidney, and MDM2, which is the negative regulator of p53, in murine fibrosarcoma and squamous cell carcinoma cell lines [79,80,81] (Figure 3). The activation of the PI3K/AKT pathway by hypoxia led to survival under hypoxic conditions and resistance to apoptosis in prostate cancer cell lines and rat pheochromocytoma cell lines [82,83]. Additionally, hypoxia inhibited the apoptotic effect of the tumor necrosis factor-related apoptosis-inducing ligand (TRAIL) by blocking the translocation of the pro-apoptotic protein BAX from the cytosol to the mitochondria in colon and lung cancer cell lines [84]. Whether hypoxia will induce apoptosis or confer resistance to apoptosis is possibly dependent on the extent of hypoxia, the cell type, and the phosphorylation status of HIF-1α, which warrants further investigation to be clarified [29,85].

In GC, human differentiated embryonic chondrocyte-expressed gene 1 (DEC1), a hypoxia-induced gene, was reported to prevent apoptosis through upregulation of Survivin in MKN45 and BGC823 GC cells. Survivin expression was higher in mouse models established from DEC1 overexpressing cells compared with the DEC1 knock-down models. Expression of DEC1 and survivin was also positively correlated in GC specimens and associated with a dismal prognosis [86]. Suppression of miR-18a was proposed as a mechanism for hypoxia-induced resistance to apoptosis in MGC-803 and HGC-27 GC cells. Hypoxia suppressed the expression of miR-18a significantly in these cell models. miR-18a mimics increased apoptosis and decreased the invasion in hypoxic conditions. Further investigation demonstrated that miR-18a leads to transcriptional suppression of HIF-1α, downregulation of BCL-2 protein, and upregulation of BAX, caspase-3, and caspase-9 proteins (Figure 3) [57].

In GC, hypoxia may also induce resistance to anoikis, a specific type of programmed cell death following the detachment of anchorage-dependent cells from the extracellular matrix. In AGS and MKN-28 GC cells, silencing or pharmacological inhibition of HIF-1α induced the expression of integrin-5 and led to anoikis [87]. In scirrhous GC cell models, activation of the FAK/SRC/PI3K/AKT and ERK signaling pathways by ANGPTL4 was proposed as a mechanism of resistance to anoikis under hypoxia. In xenograft models established from ANGPTL4 knock-down GC cells, tumorigenicity declined, and metastasis to the peritoneum diminished. Hypoxia could induce anoikis in ANGPTL4 knock-down GC cells [68]. These observations suggest a possible role of HIF-1α in facilitating the survival of metastatic GC cells in the circulation or peritoneal cavity and increasing the metastatic potential in gastric tumors.

All these findings in GC models suggest that devising HIF-1α inhibitors as a part of combination chemotherapy may have a high potential to circumvent resistance to chemotherapy-induced apoptosis and improve treatment response in GC. Comprehensive efforts to delineate precise molecular mechanisms are required to tackle this opportunity.

### 3.4. HIF-1α and Replicative Immortality in Gastric Cancer

The number of times that normal cells divide is limited due to the shortening of telomeres [88]. Cancer cells can overcome this limit by activating telomere maintenance mechanisms and gaining replicative immortality. This process differs from sustained growth factor signaling. The total number of times that a cell divide may still be limited, although the proliferation may occur without exogenous growth stimuli in the case of sustained growth factor signaling. Therefore, sustained growth factor signaling may not guarantee replicative immortality, which is highly dependent on the maintenance of the telomere length [59]. Telomerase maintains the telomere length in 85% of human tumors [89]. Human telomerase reverse transcriptase (hTERT), which is the catalytic subunit of telomerase, adds hexameric telomere repeat sequences to the 3′ ends of chromosomal DNA using the intrinsic RNA moiety human telomerase RNA (hTERC) as a template [90].

Hypoxia upregulated telomerase activity in ovarian cancer, colon cancer, cervical cancer, and renal cancer cell lines through transcriptional activation of hTERT by HIF-1α [91,92,93]. In rat gastric mucosa, telomere length increased with the increased duration of exposure to hypoxia, supported by a positive correlation between HIF-1α and hTERT expression. These observations suggested that HIF-1α may protect the gastric mucosa from lethal damage through increased telomerase activity [94].

The MAPK signaling pathway is a pivotal mediator of hypoxia-induced telomerase activity in some cancers, as shown in ovarian cancer and colon cancer cell lines [91]. Despite the limited number of studies investigating the role of hypoxia/HIF-1α in the replicative immortality of GC, the AKT pathway comes forward as the primary mediator for hypoxia-induced telomerase activity in GC (Figure 3). In AGS GC cells, treatment with the VEGFR inhibitor bevacizumab increased the expression of hTERT through the PI3K/AKT/mTOR pathway and HIF-1α [95]. In MKN28 GC cells, AKT activation upregulated hTERT. Inhibition of AKT downregulated hTERT and telomerase activity in these cells. AKT phosphorylation positively correlated with hTERT positivity and telomere length in tumor samples from 40 GC patients [96]. Additionally, visfatin, a HIF-1α-induced proinflammatory cytokine [97,98], was demonstrated to be overexpressed in AGS cells and increase hTERT expression [99]. A high perioperative plasma visfatin level was associated with poor prognosis in a study where 262 GC patients were enrolled [100]. Based on this knowledge, AKT and visfatin may be potential targets in the future to prevent HIF-1α induced replicative immortality in GC cells.

### 3.5. Induction of Angiogenesis through HIF-1α in Gastric Cancer

When tumors reach a critical size, the hypoxic microenvironment and HIFs turn on the angiogenic switch by inducing the expression of proangiogenic factors such as VEGF, VEGFRs, angiopoietins, matrix metalloproteinases (MMPs), interleukin-8, FGF, and PDGF [101] (Figure 3). Among these factors, VEGF plays a crucial role in angiogenesis. High expression of VEGF leads to tumor progression, metastasis, and poor prognosis [102]. HIF-1α is a master regulator of VEGF and angiogenesis in almost all tumors, including GC. Immunohistochemical investigation of patient specimens [103] in in vitro and in vivo studies also showed the association between HIF-1α, VEGF, and angiogenesis in GC. In TMK-1 GC cells transfected with a dominant-negative form of HIF-1α, the secretion of VEGF decreased substantially both in normoxic and hypoxic conditions. Subcutaneous or orthotopic tumor models established from TMK-1 cells with a dominant-negative form of HIF-1α were significantly smaller. The vessel maturation and density were lower compared with the HIF-1α naïve control groups [104].

Though hypoxia is the primary stimulator for HIF-1α-mediated angiogenesis, studies suggest that inflammatory mediators can also induce angiogenesis through HIF-1α. A correlation was observed between the expression of cyclooxygenase-2 (COX-2) and VEGF in GC specimens. Subsequent studies in AGS cells showed that COX-2 activity and exogenous prostaglandin E2 (PGE2) lead to stimulation of HIF-1α and VEGF expression concomitantly, suggesting the involvement of the COX-2/PGE2/HIF-1α/VEGF pathway in the induction of angiogenesis [105].

Growth factor signaling pathways also play a role in the hypoxia-independent induction of HIF-1α and angiogenesis. In a study, the expression of phosphorylated AKT (pAKT) positively correlated with HIF-1α and VEGF in 268 GC specimens. In SNU-216 and SNU-668 GC cells, constitutively active AKT (CA-AKT) induced the expression of HIF-1 protein and VEGF mRNA in a normoxic environment. However, HIF-1α and VEGF were downregulated in cells with a kinase-dead mutant of AKT. Xenograft models established from CA-AKT-expressing GC cells exhibited a higher incidence of tumor formation with larger volumes, higher micro-vessel density, and HIF-1α expression [69]. RAF1 was also reported as a stimulator of angiogenesis through HIF-1α in GC. The silencing of RAF1 down-regulated the expression of HIF-1α and VEGF in SGC7901 GC cells [106].

Growth factor receptors such as insulin-like growth factor receptors (IGFRs) also emerge as hypoxia-independent regulators of angiogenesis [107]. The blockage of IGF-1R reduced tumor angiogenesis in GC xenograft models [108]. The expression of HIF-1α and IGF2 mRNA correlated in gastric tumor samples [109]. Findings suggest that the association between IGFs and HIF-1α induction is mediated through PI3K/AKT and MAPK activation [107]. Besides these, a novel hypoxamiR, miR-382, was defined recently as an inducer of angiogenesis with possible importance as a predictive marker for progression in GC patients [110].

### 3.6. Hypoxia-Induced Epithelial-Mesenchymal Transition in Gastric Cancer

Epithelial–mesenchymal transition (EMT) is a physiological process where polarized epithelial cells adhere to the basal membrane or neighboring cells lose their polarity and gain migratory characteristics [111]. Through EMT, epithelial cancer cells transform into mesenchymal cancer cells that invade surrounding tissues and metastasize to distant sites to form new tumors [112,113]. HIF-1α induces EMT through activation of EMT-associated transcription factors TWIST, SNAIL, SLUG, SIP1, ZEB1, and MMPs, the key players in invasion and metastasis, as shown in several in vitro cancer models, including head and neck squamous carcinoma, hepatocellular carcinoma, colorectal cancer, ovarian cancer, and renal cell carcinoma cell lines [114,115,116] (Figure 3). Additionally, an increase in the expression of mesenchymal markers vimentin, fibronectin, and N-cadherin in parallel with a decrease in E-cadherin and destruction of cadherin-mediated cell–cell adhesions are critical alterations, especially for the early stages of metastasis [116].

Transforming growth factor β (TGFβ) is a critical mediator for hypoxia-induced EMT. Prolonged hypoxia (exposure to the hypoxic microenvironment for more than 10 days) induced the production of TGFβ at much higher levels compared to normoxic conditions in Lewis lung carcinoma cell lines [117]. TGFβ activates serine/threonine kinase receptors that lead to the phosphorylation of SMAD proteins in the cytoplasm. Phosphorylated SMADs activate SNAIL, ZEB1, and SIP1 in the nucleus, altering the transcription of several genes responsible for cell proliferation, differentiation, migration, and EMT. TGFβ can also induce EMT-associated transcription factors via SMAD-independent mechanisms such as MAPK, PI3K/AKT, and NF-κB signaling, as observed in in vitro models of murine mammary epithelial cells, human cervical carcinoma cells, human breast cancer cells, human kidney cells, and human salivary gland epithelial cells [118,119,120,121]. Moreover, TGFβ may cooperate with various oncogenic pathways such as NOTCH and WNT/β-catenin to trigger EMT and moderate hypoxia-induced tumor invasion and migration, as shown in prostate cancer and breast cancer cell lines [122,123,124].

The NOTCH signaling pathway also mediates hypoxia-induced EMT, besides its regulatory role in stemness, embryonic development, and cell fate. HIF-1α activated NOTCH signaling, and NOTCH1 increased HIF-1α expression in ovarian and breast cancer cell lines. Thus, once activated, NOTCH can imitate hypoxia to stimulate EMT [114]. After ligand-stimulated cleavage, intracellular domains of NOTCH translocate to the nucleus and directly increase SNAIL1 expression by binding to the SNAIL1 promoter together with other genes critical for tumor progression, as demonstrated in cervical cancer, colon cancer, ovarian cancer, and glioma cell lines. NOTCH also stabilizes SNAIL1 through increased expression of lysyl-oxidase (LOX) which protects SNAIL1 from degradation [123]. Moreover, hypoxia can increase the expression of NOTCH receptors and ligands, which stimulate a higher expression of NOTCH target genes by the accumulation of HIF-1α. Suppression of the NOTCH pathway in breast cancer cell lines decreased SLUG and SNAIL expression and blocked cellular migration, supporting the role of NOTCH signaling in hypoxia-induced EMT [124].

Emerging evidence suggests that EMT plays a substantial role in the carcinogenesis of particularly diffuse-type GC, which has a mesenchymal phenotype in contrast to intestinal GC with an epithelial phenotype [125,126]. SNAIL, ZEB, and TWIST are overexpressed in diffuse GC. Furthermore, increased TWIST expression was correlated with higher metastatic potential in GC [115]. In BGC823 and SGC7901 GC cell lines, hypoxia led to polygonal or spindle-like morphological changes and a significant increase in cell proliferation, migration, invasion, and colony formation. A decrease in E-cadherin expression and an increase in N-cadherin, vimentin, and SNAIL expression were observed in these cells. Together with EMT, hypoxia increased the expression of cancer stem cell markers SOX2, OCT4, and BMI1 bringing stem-cell characteristics to BGC823 and SGC7901 cells. Although these findings were obtained from GC cells exposed to hypoxia (1% or 5% O_2_), the mediator role of HIF-1α was not investigated in the study [31].

In hypoxia-resistant OCUM-12/Hypo cells, developed from poorly differentiated GC cell line OCUM-12, a decrease was observed in the expression of E-cadherin and zonula occludens cell adhesion molecule, while an increase in vimentin, SNAIL1, SLUG/SNAIL2, TWIST, ZEB1, ZEB, MMP-1, and MMP-2 was noted. OCUM-12/Hypo cells exhibited increased migration and invasion capability. In orthotopic tumor models, both OCUM-12 and OCUM-12/Hypo tumors were positive for HIF-1α, exhibiting heterogeneous expression throughout the tumors. OCUM-12/Hypo cells metastasized into the peritoneum and lymph nodes, whereas metastasis was not observed in orthotopic models established with OCUM-12 cells [127]. Matsuoka et al. observed that hypoxia induces EMT within 24 h in diffuse-type OCUM-2MD3 and OCUM-12 cells, which metastasize to the peritoneum. On the other hand, hypoxia did not induce EMT in OCUM-2M intestinal-type GC cells. Nevertheless, the role of HIF-1α in hypoxia-induced EMT was not investigated in OCUM-2MD3 cells [128].

In GC, evidence that supports the involvement of NOTCH signaling in hypoxia-induced EMT is almost lacking. However, there is strong evidence for the involvement of TGFβ signaling. Both TGFβ1 and TGFβR increased under hypoxic conditions in OCUM-2MD3 and OCUM-12 cells. TGFβ inhibitors repressed the induction of EMT in these cells, demonstrating the mediator role of TGFβ in hypoxia-induced EMT in diffuse-type GC. Although the HIF-1α expression increased with hypoxia in the cells, the authors stated that knocking down HIF-1α did not decrease the expression of TGFβ. Therefore, the role of HIF-1α is not clear in the process [128]. Hypoxia increased integrin expression, potentiating the implantation ability of diffuse-type GC cells to the peritoneum [129]. In SGC7901 and MKN45 (poorly differentiated) GC cell lines, the hypoxic microenvironment increased the expression of MMP-9 and urokinase-type plasminogen activator and decreased the expression of tissue inhibitor of matrix metalloproteinase (TIMP)-1, through HIF-1α-induced upregulation of 67 kDa Laminin receptor (67LR). Thus, HIF-1α increased the invasion ability of GC cells [130]. Moreover, HIF-1α upregulated chemokine receptor 4 (CXCR4), increasing the migration and invasion of KATO III GC cells [131].

Recently, the exosomal release of miR-301a-3p was suggested as an inducer of hypoxia-induced EMT, tumor progression, and metastasis in GC. In MGC803 GC cells, HIF-1α increased the exosomal release of miR-301a-3p, which targeted PDH3, decreasing the degradation of HIF-1α. Conditioned exosomes from hypoxic cells induced proliferation, EMT, invasion, migration, and colony formation in MGC803 and MKN45 cell lines. Mice treated with conditioned exosomes from hypoxic cells formed larger metastatic nodules in their lung and peritoneum. Anti-miR-301a-3p reversed these effects. Additionally, the expression of miR-301a-3p in serum exosomes of GC patients was significantly correlated with peritoneal metastasis [132].

## 4. HIF-1α and Next-Generation Hallmarks in Gastric Cancer

### 4.1. Hypoxia and Genetic Instability in Gastric Cancer

Hypoxia increases the mutation rate in mammalian cells. Mutation frequency increases further with subsequent exposures [133]. Hypoxia can induce genomic instability by gene amplification, chromosome rearrangement, and suppression of DNA mismatch repair (MMR) genes and gives rise to chemotherapy-resistant clones through microsatellite instability in several cancers, as demonstrated in breast cancer, lung cancer, and rectal cancer in vitro cell models [134,135,136,137].

**Figure 4:**
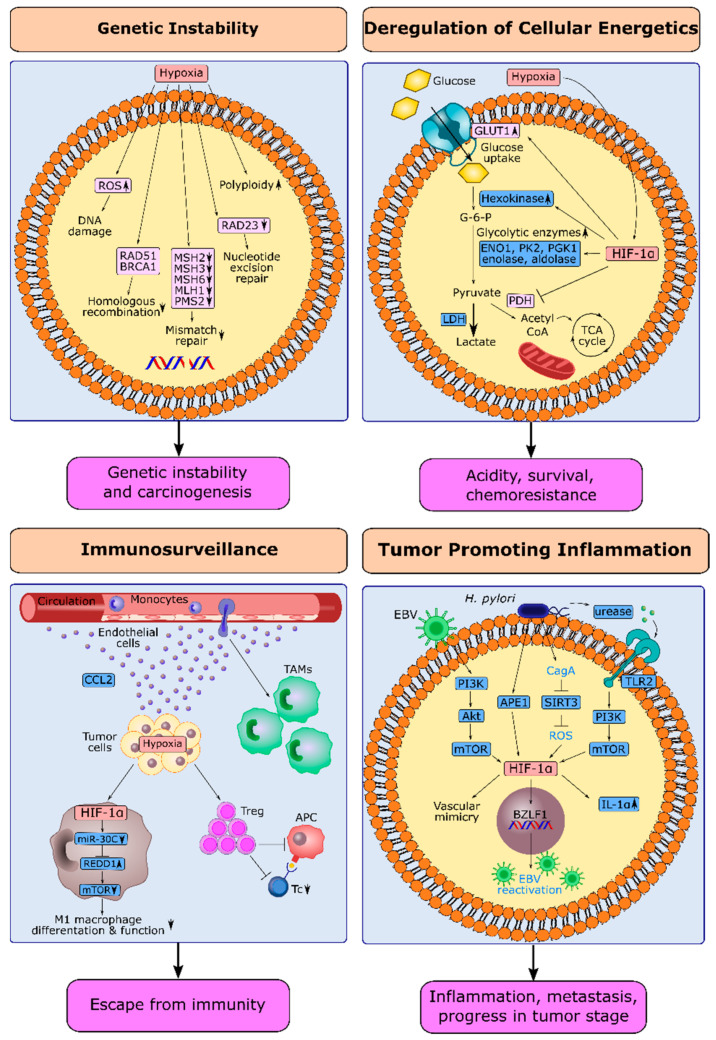
Induction of next-generation cancer hallmarks by hypoxia and HIF-1α in gastric cancer. Hypoxia induces genetic instability, deregulation in cellular energetics, escape from immune surveillance, and tumor-promoting inflammation in gastric cancer. Processes for which there is specific evidence in gastric cancer are shown in blue. Processes common in different cancers are shown in light pink. Upregulation or downregulation of specific proteins is shown with an upward or downward arrow, respectively. The figures were drawn in Inkscape 1.1.2.

ROS produced during hypoxia/reoxygenation cycles play a pivotal role in genetic instability. ROS damage DNA and generate many genomic aberrations such as base modifications, nucleotide transversions, DNA slippage mutations at microsatellites, and chromosomal-fragile sites [138]. Moreover, hypoxia suppresses DNA repair mechanisms. RAD51 and BRCA1, key actors in homologous recombination (HR), were shown to be downregulated by hypoxia, leading to impaired HR in hypoxic and post-hypoxic conditions in breast cancer, lung cancer, cervical cancer, prostate cancer, and colorectal cancer cell lines (Figure 4) [139]. Hypoxia can also suppress nucleotide excision repair through downregulation of RAD23, and MMR through downregulation of MSH2, MSH6, MSH3, MLH1, and PMS2, leading to microsatellite slippage mutations, as evidenced in lung cancer and colon cancer in vitro models [138]. Furthermore, hypoxia was proposed as an inducer of polyploidy in studies conducted with melanoma and colon cancer cell lines [140,141].

Recently, the TCGA Pan-Cancer Analysis of Whole Genomes (PCAWG) Consortium demonstrated that the extent of hypoxia positively correlates with the mutational load in whole-genome sequencing data from 1188 tumor samples of 27 different cancer types. The hypoxia score was high in 62% of the 29 GC cases investigated in the study [142]. Although extensive research investigating the impact of hypoxia on genetic instability in GC is lacking, genetic instability—either in the form of microsatellite instability (MSI) or chromosomal instability (CIN)—plays a central role in gastric carcinogenesis [143]. This may suggest hypoxia as a potential actor in gastric carcinogenesis due to its inducer role in genomic instability.

### 4.2. HIF-1α and Deregulation of Cellular Energetics in Gastric Cancer

Cellular energy metabolism is regulated dynamically, based on the energy requirements of the cell and changes in the microenvironment. Oxidative phosphorylation of glucose is the major source of energy in normal cells under normoxic conditions. Cancer cells switch from oxidative phosphorylation to oxygen-independent glycolysis and convert glucose into lactate instead of directing it to the tricarboxylic acid (TCA) cycle. This switch in cancer cells is known as the Warburg effect [144]. Though oxygen-independent glycolysis is a less efficient process in terms of ATP production than oxidative phosphorylation, it provides some advantages for the survival of cancer cells and their rapid adaptation to the microenvironment. Via glycolysis, cellular ATP requirements are met even under hypoxic conditions, and cancer cells protect themselves against the excess production of ROS that would occur during the TCA and electron transport chain (ETC) [145]. Additionally, it is a way to spare glycolysis intermediates to synthesize fatty acids, amino acids, and nucleotide precursors essential in rapidly proliferating cells [146,147].

HIF-1α has a central role in the Warburg effect [148]. HIF-1α increases the uptake of glucose into cancer cells via the transcriptional upregulation of the glucose transporter GLUT1. Thereby it provides cancer cells with higher glucose needed to keep up with cellular ATP requirements [149]. HIF-1α-induced GLUT-1 was significantly correlated with the depth of invasion, advanced stage, and shorter overall survival in GC patients [43,46]. HIF-1α increases the expression of enzymes involved in oxygen-independent glycolysis, such as enolase and aldolase [150] and inhibits the activity of pyruvate dehydrogenase required for the entry into the TCA (Figure 4) [151]. In GC cell lines, HIF-1α increased the expression of ENO1, pyruvate kinase 2, phosphoglycerate kinase 1, and lactate dehydrogenase A (LDHA), critical enzymes in the glycolytic pathway [152]. HIF-1 transcriptionally up-regulates hexokinase (HK), the catalyzer of the first reaction in glycolysis. HK2 was overexpressed in 16.7% of the GC specimens. HK2 expression was inversely related to BCL-2 expression and associated with poor survival [153]. HIF-1α-induced glycolysis was also suggested as a mechanism for hypoxia-induced 5-fluorouracil resistance in GC cells [154].

### 4.3. HIF-1α and Escape from Immune Surveillance in Gastric Cancer

Cancer cells are in continuous interaction with the immune system and utilize several strategies to evade anti-tumor immunity. Hypoxia plays a reinforcing role in these strategies [155]. Hypoxic tumor niches are enriched in tumor-associated macrophages (TAMs), which suppress anti-tumor immunity and induce tumor progression [156]. Monocytes recruited to tumors differentiate into TAMs and secrete growth factors, proangiogenic factors, and immunosuppressive mediators under the influence of HIFs [157]. In GC specimens, the expression of HIF-1α was correlated with monocyte chemoattractant protein-1, which plays a critical role in the recruitment of monocytes and macrophages to tumors (Figure 4) [158]. A significant correlation was found between the level of hypoxia and the number of TAMs in GC specimens of 105 patients. The risk of death was two-fold in patients whose tumors had moderate hypoxia and large numbers of TAMs, whereas the number of TAMs itself did not impact the risk of death if the tumors were mildly hypoxic [159]. The results of this study were supported by another study where HIF-1α expression showed a significant correlation with TAM infiltration in tumor biopsies from 236 GC patients. A concomitant increase in HIF-1α expression and TAMs in tumor tissue was identified as an independent risk factor for poor prognosis [160]. TAMs increased the invasion and migration rate of AGS, HGC-27, Hs-746T, and NCI-N87 GC cells. Hypoxia further strengthened the impact of TAMs on these cell lines [161].

In GC-infiltrating macrophages, HIF-1α suppressed miR-30c, increasing the expression of REDD1, an inhibitor of mTOR. Consequently, the concomitant decreases in mTOR activity and glycolysis led to the inhibition of M1 macrophage differentiation and function. Since M1 macrophages have cytotoxic activity on cancer cells, the HIF-1α/miR-30c/REDD1/mTOR axis was suggested as a mechanism for hypoxia-induced suppression of anti-tumor immunity in GC (Figure 4) [162]. Dysregulation of cellular energetics may also be a mechanism by which hypoxia impairs anti-tumor immunity. Hypoxia-induced down-regulation of miR-34a increased the expression of LDHA, which increases lactate production in GC tumor-infiltrating lymphocytes. A high lactate concentration was negatively correlated with the number of pro-inflammatory Th1 cells and cytotoxic T lymphocytes (CTLs) in GC specimens [163]. Suppression of CTLs and antigen-presenting cells via regulatory T (Treg) cells is another important strategy for tumor cells to evade anti-tumor immunity [164]. HIF-1α expression was correlated with the number of Treg cells in GC specimens. This correlation was stronger in metastatic tumors compared to non-metastatic tumors. Hypoxia-induced upregulation of TGF-β was suggested as a mechanism for the expansion of Treg cells under hypoxia in GC [165].

### 4.4. HIF-1α and Tumor-Promoting Inflammation in Gastric Cancer

The inflammatory tumor microenvironment is a critical hallmark for carcinogenesis and tumor progression that facilitates the acquisition of other hallmark capabilities via the release of GFs, proangiogenic mediators, proteases, ROS, and EMT-activating signals [59,166]. Pro-inflammatory cytokine interleukin-1α (IL-1α) increases in several malignancies, including GC. Increased IL-1α expression is significantly associated with liver metastasis, lymph node metastasis, increased tumor stage, and decreased survival in GC patients [167,168]. Hypoxia increased IL-1α expression via HIF-1α in GC cell lines [168].

Bacterial or viral infections precede chronic inflammation in approximately 20% of all cancers [166]. GC associated with *H. pylori* or Epstein–Barr virus (EBV) is among the most prominent examples of tumor-promoting inflammation in cancer [166,169]. Chronic inflammation induced by *H. pylori* in gastric epithelium promotes a sequence of pathologies from gastritis to intestinal-type GC [170]. Additionally, *H. pylori* infection is associated with MALT lymphoma [166]. Based on the molecular classification of GC, 9% of GCs are associated with EBV infection, which constitutes a distinct molecular subtype [171].

Emerging evidence suggests that HIF-1α is involved in GC, promoting inflammation induced by *H. pylori* and EBV [172]. HIF-1α increases significantly in patients with *H. pylori*-positive gastritis, compared with *H. pylori*-negative gastritis [173]. The ROS and NO generated in *H. pylori*-infected gastric epithelium inhibit the hydroxylation and degradation of HIF-1α even in normoxic conditions [174]. *H. pylori* oncoprotein CagA downregulates sirtuin 3 (SIRT3), a tumor-suppressor protein that suppresses ROS production. Thereby, CagA increases the expression of HIF-1α and its target genes (Figure 4) [175]. Additionally, urease secreted by *H. pylori* activates PI3K/mTOR signaling and induces HIF-1α expression, through binding to toll-like receptor 2 on gastric cells [176]. Moreover, induction of apurinic/apyrimidinic endonuclease 1 (APE1) in *H. pylori*-infected gastric epithelium increases the binding of APE1 to transcriptional coactivator p300 and increases HIF-1α expression [177].

Investigation of *H. pylori*-positive gastritis specimens demonstrated that HIF-1α expression particularly increases in areas infiltrated by macrophages. Bone marrow-derived macrophages also exhibited high HIF-1α expression in mice models infected with *H. pylori*. *H. pylori*-induced HIF-1α expression is strongly associated with increased pro-inflammatory cytokines IL-1β and IL-6, and NO synthase in vivo. Surprisingly, an increase in systemic inflammation and aggravation in gastritis was observed in *H. pylori*-infected transgenic mice where HIF-1α is inactivated specifically in the myeloid cell lineage. These observations suggested that a HIF-1α increase in macrophages may contribute to the bactericidal action, in contrast to the contribution of HIF-1α to tumor-promoting inflammation in *H. pylori*-infected gastric epithelium [178].

HIF-1α is also involved in the natural life cycle of EBV and lytic infection that induce tumorigenesis. HIF-1α stabilizers increased the EBV lytic proteins and reactivated EBV infection in EBV-positive GC cell lines. HIF-1α induced this response via the direct binding and activation of the EBV primary latent-lytic switch BZLF1 gene, Zp (Figure 4) [179]. Additionally, the PI3K/AKT/mTOR/HIF-1α axis has been put forth as a mediator of EBV-induced vascular mimicry (VM). VM defines channel-like structures established by tumor cells that mimic the vasculature and contribute substantially to tumor progression and metastasis under hypoxic conditions. VM formation is observed in EBV-positive GC cells, while EBV-negative GC cells do not exhibit VM. Moreover, HIF-1α expression and AKT phosphorylation were correlated with VM formation in tumor samples from EBV-associated GC patients [180].

## 5. Conclusions

HIF-1α is in a complex interplay with all hallmarks of cancer. There is substantial evidence that HIF-1α plays a pivotal role in hypoxia-induced angiogenesis, the Warburg effect, and resistance to apoptosis in GC. The impact of HIF-1α on these hallmarks can be of critical importance in GC progression and chemoresistance. Additionally, HIF-1α stands out as a mediator of tumor-promoting inflammation in *H. pylori* and EBV-associated GCs, implying its involvement in gastric carcinogenesis. However, further studies are needed to elucidate underlying mechanisms in full detail. The knowledge gap is even further for the role of HIF-1α in sustained GF signaling, EMT, evasion from growth suppressors, genetic instability, and evasion from immunosurveillance in GC. Investigation of these mechanisms may reveal critical links between HIF-1α and these hallmarks in GC.

The GFRs that might be upregulated or activated by HIF-1α are currently unknown in GC. Genomic and proteomic profiling studies in GC cell lines after exposure to hypoxia or overexpression of HIF-1α may be an efficient first step hereof. The expression of candidate GFRs identified from these in vitro studies can be investigated in tumor specimens for their overlap with hypoxic niches and further tested in animal models. Identifying the HIF-1α-induced GFRs would provide a great opportunity to inhibit HIF-1α-induced oncogenic pathways in GC.

Among oncogenic signaling pathways, the PI3K/AKT pathway may have a predominant role in mediating the effects of HIF-1α in GC. There is evidence for its mediator role in hypoxia-induced resistance to anoikis, replicative immortality, angiogenesis, and tumor-promoting inflammation in GC [68,95,96,176,180]. Therefore, targeting the PI3K/AKT pathway may be a strategy to antagonize the action of HIF-1α or synergize with HIF-1α inhibitors in GC. Agents that target the PI3K/AKT pathway may also prevent the transformation of premalignant gastric lesions into full malignancy, considering the mediator role of the PI3K/AKT/mTOR/HIF-1α axis in tumor-promoting inflammation in *H. pylori*- and EBV-associated GC [176,180]. Further studies in GC specimens and animal models should be designed accordingly. The development of 3D tumor models to observe the transformation of pre-malignant cells into cancer cells would also be a profound advancement for exploring this opportunity.

An important point to mention here is the redundancy of signaling pathways and oncogenic bypass, which are central to the emergence of resistance to molecular-targeted agents in cancer cells [181]. The role of other hypoxia-induced signaling pathways such as the MAPK pathway, which may be redundant to the PI3K/AKT pathway, was not dissected well in GC. Short-term exposure of GC cell lines to hypoxia will give only limited information about redundant pathways. Thus, investigating these mechanisms in GC cell models under long-term exposure to hypoxia is required to attain sufficient data on the redundancy of HIF-1- induced oncogenic pathways, which may uncover more effective strategies to inhibit HIF-1α signaling.

Besides AKT, ANGLPTL4 and visfatin may be important targets to prevent HIF-1α-induced resistance to apoptosis and replicative immortality, respectively. On the other hand, melatonin, mimics of miR-18a or miR-186, and increasing the activity of PHD2, PHD3, or RUNX3 may be more direct strategies to downregulate HIF-1α. However, the number of studies is limited for precisely predicting their action. Meticulous in vitro and in vivo studies should be performed to comprehend their precise roles in the regulation of HIF-1α.

One important caveat for the inhibition or down-regulation of HIF-1α is the observation that cancer cells selected under hypoxia and expressing high levels of HIF-1α may mostly have loss-of-function mutations in p53, which will render them resistant to the apoptotic effect of any intervention [78]. The p53 mutation status of hypoxic cells in gastric tumors is unclear, which should be examined to assess the benefit of HIF-1α-inhibiting strategies in GC patients. Additionally, the impact of HIF-1α on genomic instability in GC is not known. Genomic alterations induced by hypoxia such as base modifications, DNA slippage mutations at microsatellites, and chromosomal-fragile sites should be demonstrated in GC cells.

One of the biggest gaps in the impact of HIF-1α on GC may be hypoxia-induced EMT. Though evidence suggests that hypoxia is an inducer of EMT in GC cells, the involvement of HIF-1α in this process is not delineated well [128]. New in vitro and in vivo studies should address the significance of HIF-1α in hypoxia-induced EMT.

Last but not the least, HIF-1α may be a contributing factor to the poor response to angiogenesis inhibitors or immunotherapies. Increased HIF-1α expression induced by anti-angiogenic agents may contribute to increased aggressiveness of cancer cells at hypoxic niches [182]. This may limit the benefit of anti-angiogenic agents such as ramucirumab in GC. Since high levels of hypoxia accompanying TAM infiltration was observed to increase the aggressiveness of GC cell lines [161] and HIF-1α expression was correlated with the number of Treg in GC specimens [165], HIF-1α activity may also be a limiting factor for the efficacy of immunotherapies in GC patients. Hence, investigation of the precise role of HIF-1α in GC is not only required to develop new treatment strategies but may also expose new ways to increase the efficacy of currently available therapeutics in GC.

## Data Availability

Publicly available ClinicalTrials.gov database was searched in this study to compile the number of clinical trials started each year for the indication of cancer which mention “hypoxia” or “hypoxia inducible factor”. This database can be reached here: https://clinicaltrials.gov.

## Abbreviations

67LR: 67 kDa laminin receptor, ADM: adrenomedullin, AKT: protein kinase B, ANGPTL4: angiopoietin like-4 protein, APAF1: apoptotic protease activating factor 1, APC: antigen-presenting cell, APE1: apurinic/apyrimidinic endonuclease 1, ATP: adenosine triphosphate, BAX: Bcl-2-associated X protein, BCL-2: anti-apoptotic protein B-cell lymphoma 2, BMI1: B-cell-specific Moloney murine leukemia virus integration site-1, BNIP3: BCL2/adenovirus EIB 19 kD-interacting protein 3, BRCA1: breast cancer type 1 susceptibility protein, β-catenin: catenin beta-1 protein, CA-AKT: constitutively active AKT, Caspase: cysteine-aspartic protease, CBP: cyclic AMP response element-binding protein, CCL2: monocyte chemotactic protein-1, CIN: chromosomal instability, c-met: tyrosine-protein kinase Met, COX-2: cyclooxygenase-2, CXCR4: chemokine receptor 4, Cyto c: cytochrome c, DEC1: differentiated embryonic chondrocyte-expressed gene 1, EBV: Epstein–Barr virus, EDN1: endothelin1, EGF: epidermal growth factor, EGFR: epidermal growth factor receptor, EMT: epithelial–mesenchymal transition, ENO: enolase, ENO1: enolase 1, EPO: erythropoietin, ERK: extracellular-signal-regulated kinase, ETC: electron transport chain, FAK: focal adhesion kinase, FGF: fibroblast growth factor, FGFR1: fibroblast growth factor receptor 1, FOXO1: mammalian forkhead transcription factor of the O class 1, G-6-P: glucose-6-phosphatase, GC: gastric cancer, GF: growth factor, GFR: growth factor receptor, GLUT1: glucose transporter, *H. pylori*: *Helicobacter pylori*, HIF: hypoxia-inducible factor, HIF-1α: hypoxia-inducible factor 1α, HIF-1β: hypoxia-inducible factor 1β, HIF-2: hypoxia-inducible factor 2, HIF-2α: hypoxia-inducible factor 2α, HIF-3α: hypoxia-inducible factor 3α, HR: homologous recombination, HRE: hypoxia-response elements, hTERC: human telomerase RNA, hTERT: human telomerase reverse transcriptase, IAP-2: inhibitor of apoptosis protein 2, IGF: insulin-like growth factor, IGF1: insulin-like growth factor 1, IGF-1R: insulin-like growth factor type 1 receptor, IGF2: insulin-like growth factor 2, IGFR: insulin-like growth factor receptor, IL: interleukin, JNK: c-Jun NH2-terminal kinase, LDH: lactate dehydrogenase, LDHA: lactate dehydrogenase A, LOX: lysyl-oxidase, MAPK: mitogen-activated protein kinase, MDM2: mouse double minute 2 homolog protein, miR: microRNA, MLH: MutL homolog, MLH1: MutL homolog 1, MMP: matrix metalloproteinase, MMP-1: matrix metalloproteinase 1, MMP-2: matrix metalloproteinase 2, MMP-9: matrix metalloproteinase 9, MMR: DNA mismatch repair, mRNA: messenger RNA, MSH: MutS homolog, MSH2: MutS homolog 2, MSH3: MutS homolog 3, MSH6: MutS homolog 6, MSI: microsatellite instability, mTOR: mammalian target of rapamycin, NF-κB: nuclear factor-kappa B, NIX: Nip3-like protein X, NMR spectroscopy: nuclear magnetic resonance spectroscopy, NO: nitric oxide, NOS: nitric oxide species, NOTCH1: NOTCH homolog 1, OCT4: octamer-binding transcription factor 4, p27: cyclin-dependent kinase inhibitor, p300: transcriptional coactivator p300, p53: tumor protein p53, pAKT: phosphorylated AKT, PDGF: platelet-derived growth factor, PDH: pyruvate dehydrogenase, PGE2: prostaglandin E2, PGK1: phosphoglycerate kinase 1, PHD: prolyl hydroxylase, PI3K: phosphoinositide 3-kinase, PK2: pyruvate kinase 2, PMS2: Mismatch repair endonuclease PMS2, PTEN: Phosphatase and tensin homolog, pVHL: von Hippel–Lindau tumor suppressor protein, RAD23: UV excision repair protein RAD23, RAD51: DNA repair protein RAD51 homolog 1, RAF1: RAF proto-oncogene serine/threonine-protein kinase, RAS: Ras GTPase, REDD1: regulated in development and DNA damage responses 1, RON: recepteur d’origine Nantais, ROS: reactive oxygen species, RUNX3: runt-related transcription factor 3, RZR/RORγ: melatonin nuclear receptor RZR/RORγ, SIP1: Smad-interacting protein 1, SIRT3: sirtuin 3, SLUG: zinc finger protein SNAI2, SNAIL: zinc finger protein SNAI1, SOX2: SRY (sex determining region Y)-box 2, SRC: Src kinase, TAM: tumor-associated macrophage, Tc: cytotoxic T cell, TCA cycle: tricarboxylic acid (TCA) cycle, TGF-α: transforming growth factor-α, TGFβ: transforming growth factor β, TGF-β1: transforming growth factor beta 1, TGFβR: transforming growth factor beta receptor, Th cell: helper T cell, TIMP-1: tissue inhibitor of matrix metalloproteinase 1, TLR: toll-like receptor, TNM: TNM Classification of Malignant Tumors, TRAIL: tumor necrosis factor-related apoptosis inducing ligand, Treg: regulatory T cell, Ub: ubiquitin tag, uPA: urokinase-type plasminogen activator, VEGF: vascular endothelial growth factor, VEGFR: VEGF receptor, VEGFR1: VEGF receptor 1, VHL: Von Hippel–Lindau tumor suppressor, VM: vascular mimicry, WNT: wingless-related integration site, ZEB1: zinc finger E-box-binding homeobox protein 1.

## References

[1] Sung H., Ferlay J., Siegel R.L., Laversanne M., Soerjomataram I., Jemal A., Bray F. (2021). Global Cancer Statistics 2020: GLOBOCAN Estimates of Incidence and Mortality Worldwide for 36 Cancers in 185 Countries. CA Cancer J. Clin..

[2] Smyth E.C., Nilsson M., Grabsch H.I., van Grieken N.C., Lordick F. (2020). Gastric cancer. Lancet.

[3] Ajani J.A., Lee J., Sano T., Janjigian Y.Y., Fan D., Song S. (2017). Gastric adenocarcinoma. Nat. Rev. Dis. Primers.

[4] Smyth E.C., Verheij M., Allum W., Cunningham D., Cervantes A., Arnold D., Committee E.G. (2016). Gastric cancer: ESMO Clinical Practice Guidelines for diagnosis, treatment and follow-up. Ann. Oncol..

[5] Ajani J.A., D’Amico T.A., Bentrem D.J., Chao J., Cooke D., Corvera C., Das P., Enzinger P.C., Enzler T., Fanta P. (2022). Gastric Cancer, Version 2.2022, NCCN Clinical Practice Guidelines in Oncology. J. Natl. Compr. Cancer Netw..

[6] Ruan T., Liu W., Tao K., Wu C. (2020). A Review of Research Progress in Multidrug-Resistance Mechanisms in Gastric Cancer. Onco Targets Ther..

[7] Shitara K., Özgüroğlu M., Bang Y.-J., Di Bartolomeo M., Mandalà M., Ryu M.-H., Fornaro L., Olesiński T., Caglevic C., Chung H.C. (2018). Pembrolizumab versus paclitaxel for previously treated, advanced gastric or gastro-oesophageal junction cancer (KEYNOTE-061): A randomised, open-label, controlled, phase 3 trial. Lancet.

[8] Shitara K., Ajani J.A., Moehler M., Garrido M., Gallardo C., Shen L., Yamaguchi K., Wyrwicz L., Skoczylas T., Bragagnoli A.C. (2022). Nivolumab plus chemotherapy or ipilimumab in gastro-oesophageal cancer. Nature.

[9] Janjigian Y.Y., Shitara K., Moehler M., Garrido M., Salman P., Shen L., Wyrwicz L., Yamaguchi K., Skoczylas T., Campos Bragagnoli A. (2021). First-line nivolumab plus chemotherapy versus chemotherapy alone for advanced gastric, gastro-oesophageal junction, and oesophageal adenocarcinoma (CheckMate 649): A randomised, open-label, phase 3 trial. Lancet.

[10] Kelly R.J., Ajani J.A., Kuzdzal J., Zander T., Van Cutsem E., Piessen G., Mendez G., Feliciano J., Motoyama S., Lièvre A. (2021). Adjuvant Nivolumab in Resected Esophageal or Gastroesophageal Junction Cancer. N. Engl. J. Med..

[11] Janjigian Y.Y., Kawazoe A., Yañez P., Li N., Lonardi S., Kolesnik O., Barajas O., Bai Y., Shen L., Tang Y. (2021). The KEYNOTE-811 trial of dual PD-1 and HER2 blockade in HER2-positive gastric cancer. Nature.

[12] Tabernero J., Hoff P.M., Shen L., Ohtsu A., Shah M.A., Cheng K., Song C., Wu H., Eng-Wong J., Kim K. (2018). Pertuzumab plus trastuzumab and chemotherapy for HER2-positive metastatic gastric or gastro-oesophageal junction cancer (JACOB): Final analysis of a double-blind, randomised, placebo-controlled phase 3 study. Lancet Oncol..

[13] Bang Y.-J., Van Cutsem E., Feyereislova A., Chung H.C., Shen L., Sawaki A., Lordick F., Ohtsu A., Omuro Y., Satoh T. (2010). Trastuzumab in combination with chemotherapy versus chemotherapy alone for treatment of HER2-positive advanced gastric or gastro-oesophageal junction cancer (ToGA): A phase 3, open-label, randomised controlled trial. Lancet.

[14] Kawazoe A., Fukuoka S., Nakamura Y., Kuboki Y., Wakabayashi M., Nomura S., Mikamoto Y., Shima H., Fujishiro N., Higuchi T. (2020). Lenvatinib plus pembrolizumab in patients with advanced gastric cancer in the first-line or second-line setting (EPOC1706): An open-label, single-arm, phase 2 trial. Lancet Oncol..

[15] Etemadi A., Safiri S., Sepanlou S.G., Ikuta K., Bisignano C., Shakeri R., Amani M., Fitzmaurice C., Nixon M., Abbasi N. (2020). The global, regional, and national burden of stomach cancer in 195 countries, 1990–2017: A systematic analysis for the Global Burden of Disease study 2017. Lancet Gastroenterol. Hepatol..

[16] Joshi S.S., Badgwell B.D. (2021). Current treatment and recent progress in gastric cancer. CA Cancer J. Clin..

[17] Morgan E., Arnold M., Camargo M.C., Gini A., Kunzmann A.T., Matsuda T., Meheus F., Verhoeven R.H.A., Vignat J., Laversanne M. (2022). The current and future incidence and mortality of gastric cancer in 185 countries, 2020–40: A population-based modelling study. eClinicalMedicine.

[18] Senthebane D.A., Rowe A., Thomford N.E., Shipanga H., Munro D., Al Mazeedi M.A., Almazyadi H.A., Kallmeyer K., Dandara C., Pepper M.S. (2017). The Role of Tumor Microenvironment in Chemoresistance: To Survive, Keep Your Enemies Closer. Int. J. Mol. Sci..

[19] Rihawi K., Ricci A.D., Rizzo A., Brocchi S., Marasco G., Pastore L.V., Llimpe F.L.R., Golfieri R., Renzulli M. (2021). Tumor-Associated Macrophages and Inflammatory Microenvironment in Gastric Cancer: Novel Translational Implications. Int. J. Mol. Sci..

[20] Moreira A.M., Pereira J., Melo S., Fernandes M.S., Carneiro P., Seruca R., Figueiredo J. (2020). The Extracellular Matrix: An Accomplice in Gastric Cancer Development and Progression. Cells.

[21] Rybinski B., Yun K. (2016). Addressing intra-tumoral heterogeneity and therapy resistance. Oncotarget.

[22] Trédan O., Galmarini C.M., Patel K., Tannock I.F. (2007). Drug Resistance and the Solid Tumor Microenvironment. J. Natl. Cancer Inst..

[23] Miao Z.-F., Wang Z.-N., Zhao T.-T., Xu Y.-Y., Gao J., Miao F., Xu H.-M. (2014). Peritoneal Milky Spots Serve as a Hypoxic Niche and Favor Gastric Cancer Stem/Progenitor Cell Peritoneal Dissemination through Hypoxia-Inducible Factor 1α. Stem Cells.

[24] Chen L., Shi Y., Yuan J., Han Y., Qin R., Wu Q., Jia B., Wei B., Wei L., Dai G. (2014). HIF-1 Alpha Overexpression Correlates with Poor Overall Survival and Disease-Free Survival in Gastric Cancer Patients Post-Gastrectomy. PLoS ONE.

[25] Bubnovskaya L., Kovelskaya A., Gumenyuk L., Ganusevich I., Mamontova L., Mikhailenko V., Osinsky D., Merentsev S., Osinsky S. (2014). Disseminated Tumor Cells in Bone Marrow of Gastric Cancer Patients: Correlation with Tumor Hypoxia and Clinical Relevance. J. Oncol..

[26] Bubnovskaya L., Osinsky D. (2020). Tumor microenvironment and metabolic factors: Contribution to gastric cancer. Exp. Oncol..

[27] Bubnovskaya L., Osinsky D., Trachevsky V., Naleskina L., Kovelskaya A., Gumenyuk L. (2014). Premorphological alterations in gastric mucosa in patients with gastric cancer: Hypoxia level assessed by 31P NMR spectroscopy. Exp. Oncol..

[28] Eales K.L., Hollinshead K.E.R., Tennant D.A. (2016). Hypoxia and metabolic adaptation of cancer cells. Oncogenesis.

[29] Ruan K., Song G., Ouyang G. (2009). Role of hypoxia in the hallmarks of human cancer. J. Cell. Biochem..

[30] Vaupel P. (2008). Hypoxia and Aggressive Tumor Phenotype: Implications for Therapy and Prognosis. Oncologist.

[31] Guo J., Wang B., Fu Z., Wei J., Lu W. (2016). Hypoxic Microenvironment Induces EMT and Upgrades Stem-Like Properties of Gastric Cancer Cells. Technol. Cancer Res. Treat..

[32] Huang L., Wu R.L., Xu A.M. (2015). Epithelial-mesenchymal transition in gastric cancer. Am. J. Transl. Res..

[33] Shirai Y., Chow C.C., Kambe G., Suwa T., Kobayashi M., Takahashi I., Harada H., Nam J.-M. (2021). An Overview of the Recent Development of Anticancer Agents Targeting the HIF-1 Transcription Factor. Cancers.

[34] Patnaik A., Chiorean E.G., Tolcher A., Papadopoulos K., Beeram M., Kee D., Waddell M., Gilles E., Buchbinder A. (2009). EZN-2968, a novel hypoxia-inducible factor-1α (HIF-1α) messenger ribonucleic acid (mRNA) antagonist: Results of a phase I, pharmacokinetic (PK), dose-escalation study of daily administration in patients (pts) with advanced malignancies. J. Clin. Oncol..

[35] Jeong W., Rapisarda A., Park S.R., Kinders R.J., Chen A., Melillo G., Turkbey B., Steinberg S.M., Choyke P., Doroshow J.H. (2014). Pilot trial of EZN-2968, an antisense oligonucleotide inhibitor of hypoxia-inducible factor-1 alpha (HIF-1α), in patients with refractory solid tumors. Cancer Chemother. Pharmacol..

[36] Wu J., Contratto M., Shanbhogue K.P., Manji G.A., O’Neil B.H., Noonan A., Tudor R., Lee R. (2019). Evaluation of a locked nucleic acid form of antisense oligo targeting HIF-1α in advanced hepatocellular carcinoma. World J. Clin. Oncol..

[37] Masoud G.N., Li W. (2015). HIF-1α pathway: Role, regulation and intervention for cancer therapy. Acta Pharm. Sin. B.

[38] Fallah J., Rini B.I. (2019). HIF Inhibitors: Status of Current Clinical Development. Curr. Oncol. Rep..

[39] Semenza G.L. (2012). Hypoxia-Inducible Factors in Physiology and Medicine. Cell.

[40] Kaelin W.G., Ratcliffe P.J. (2008). Oxygen Sensing by Metazoans: The Central Role of the HIF Hydroxylase Pathway. Mol. Cell.

[41] Kitajima Y., Miyazaki K. (2013). The Critical Impact of HIF-1a on Gastric Cancer Biology. Cancers.

[42] Lin S., Ma R., Zheng X.-Y., Yu H., Liang X., Lin H., Cai X.-J. (2014). Meta-analysis of immunohistochemical expression of hypoxia inducible factor-1α as a prognostic role in gastric cancer. World J. Gastroenterol..

[43] Hao L.S., Liu Q., Tian C., Zhang D.X., Wang B., Zhou D.X., Li Z.P., Yuan Z.X. (2019). Correlation and expression analysis of hypoxia-inducible factor 1α, glucose transporter 1 and lactate dehydrogenase 5 in human gastric cancer. Oncol. Lett..

[44] Rohwer N., Lobitz S., Daskalow K., Jöns T., Vieth M., Schlag P.M., Kemmner W., Wiedenmann B., Cramer T., Höcker M. (2009). HIF-1α determines the metastatic potential of gastric cancer cells. Br. J. Cancer.

[45] Griffiths E.A., Pritchard S.A., Valentine H.R., Whitchelo N., Bishop P.W., Ebert M.P., Price P.M., Welch I.M., West C.M. (2007). Hypoxia-inducible factor-1α expression in the gastric carcinogenesis sequence and its prognostic role in gastric and gastro-oesophageal adenocarcinomas. Br. J. Cancer.

[46] Jung J.-H., Im S., Jung E.S., Kang C.S. (2013). Clinicopathological Implications of the Expression of Hypoxia-related Proteins in Gastric Cancer. Int. J. Med. Sci..

[47] Ma J., Zhang L., Ru G.Q., Zhao Z.S., Xu W.J. (2007). Upregulation of hypoxia inducible factor 1α mRNA is associated with elevated vascular endothelial growth factor expression and excessive angiogenesis and predicts a poor prognosis in gastric carcinoma. World J. Gastroenterol..

[48] Muz B., de la Puente P., Azab F., Azab A.K. (2015). The role of hypoxia in cancer progression, angiogenesis, metastasis, and resistance to therapy. Hypoxia.

[49] Song M.S., Salmena L., Pandolfi P.P. (2012). The functions and regulation of the PTEN tumour suppressor. Nat. Rev. Mol. Cell Biol..

[50] Ravi R., Mookerjee B., Bhujwalla Z.M., Sutter C.H., Artemov D., Zeng Q., Dillehay L.E., Madan A., Semenza G.L., Bedi A. (2000). Regulation of tumor angiogenesis by p53-induced degradation of hypoxia-inducible factor 1α. Genes Dev..

[51] Kamphues C., Wittschieber D., Klauschen F., Kasajima A., Dietel M., Schmidt S.C., Glanemann M., Bahra M., Neuhaus P., Weichert W. (2012). Prolyl Hydroxylase Domain 2 Protein Is a Strong Prognostic Marker in Human Gastric Cancer. Pathobiology.

[52] Xia Y.J., Jiang X.T., Jiang S.B., He X.J., Luo J.G., Liu Z.C., Wang L., Tao H.Q., Chen J.Z. (2017). PHD3 affects gastric cancer progression by negatively regulating HIF1A. Mol. Med. Rep..

[53] Lee S.H., Bae S.C., Kim K.W., Lee Y.M. (2014). RUNX3 inhibits hypoxia-inducible factor-1α protein stability by interacting with prolyl hydroxylases in gastric cancer cells. Oncogene.

[54] Liu B., Han Y., Jiang L., Jiang D., Li W., Zhang T., Zu G., Zhang X. (2018). Clinicopathological and prognostic significance of the RUNX3 expression in gastric cancer: A systematic review and meta-analysis. Int. J. Surg..

[55] Kim S.Y., Ko Y.S., Park J., Choi Y., Park J.W., Kim Y., Pyo J.S., Yoo Y.B., Lee J.-S., Lee B.L. (2016). Forkhead Transcription Factor FOXO1 Inhibits Angiogenesis in Gastric Cancer in Relation to SIRT1. Cancer Res. Treat..

[56] Wang R.-X., Liu H., Xu L., Zhang H., Zhou R.-X. (2015). Involvement of nuclear receptor RZR/RORγ in melatonin-induced HIF-1α inactivation in SGC-7901 human gastric cancer cells. Oncol. Rep..

[57] Wu F., Huang W., Wang X. (2015). microRNA-18a regulates gastric carcinoma cell apoptosis and invasion by suppressing hypoxia-inducible factor-1α expression. Exp. Ther. Med..

[58] Cho H.S., Han T.S., Hur K., Ban H.S. (2019). The Roles of Hypoxia-Inducible Factors and Non-Coding RNAs in Gastrointestinal Cancer. Genes.

[59] Hanahan D., Weinberg R.A. (2011). Hallmarks of cancer: The next generation. Cell.

[60] Powis G., Kirkpatrick L. (2014). Hypoxia inducible factor-1alpha as a cancer drug target. Mol Cancer Ther..

[61] Semenza G.L. (2012). Hypoxia-inducible factors: Mediators of cancer progression and targets for cancer therapy. Trends Pharmacol. Sci..

[62] Lu Y., Liu Y., Oeck S., Zhang G.J., Schramm A., Glazer P.M. (2020). Hypoxia Induces Resistance to EGFR Inhibitors in Lung Cancer Cells via Upregulation of FGFR1 and the MAPK Pathway. Cancer Res..

[63] Waltenberger J., Mayr U., Pentz S., Hombach V. (1996). Functional Upregulation of the Vascular Endothelial Growth Factor Receptor KDR by Hypoxia. Circulation.

[64] Wang Y., Roche O., Xu C., Moriyama E.H., Heir P., Chung J., Roos F.C., Chen Y., Finak G., Milosevic M. (2012). Hypoxia promotes ligand-independent EGF receptor signaling via hypoxia-inducible factor–mediated upregulation of caveolin-1. Proc. Natl. Acad. Sci. USA.

[65] Li X., Lu Y., Liang K., Pan T., Mendelsohn J., Fan Z. (2008). Requirement of hypoxia-inducible factor-1α down-regulation in mediating the antitumor activity of the anti–epidermal growth factor receptor monoclonal antibody cetuximab. Mol. Cancer Ther..

[66] Zhou D., Huang L., Zhou Y., Wei T., Yang L., Li C. (2019). RON and RONΔ160 promote gastric cancer cell proliferation, migration, and adaption to hypoxia via interaction with β-catenin. Aging.

[67] Zhang J., Xu J., Dong Y., Huang B. (2018). Down-regulation of HIF-1α inhibits the proliferation, migration, and invasion of gastric cancer by inhibiting PI3K/AKT pathway and VEGF expression. Biosci. Rep..

[68] Baba K., Kitajima Y., Miyake S., Nakamura J., Wakiyama K., Sato H., Okuyama K., Kitagawa H., Tanaka T., Hiraki M. (2017). Hypoxia-induced ANGPTL4 sustains tumour growth and anoikis resistance through different mechanisms in scirrhous gastric cancer cell lines. Sci. Rep..

[69] Lee B.L., Kim W.H., Jung J., Cho S.J., Park J.-W., Kim J., Chung H.-Y., Chang M.S., Nam S.Y. (2008). A hypoxia-independent up-regulation of hypoxia-inducible factor-1 by AKT contributes to angiogenesis in human gastric cancer. Carcinogenesis.

[70] Tamura G. (2006). Alterations of tumor suppressor and tumor-related genes in the development and progression of gastric cancer. World J. Gastroenterol..

[71] Yang L., Kuang L.G., Zheng H.C., Li J.Y., Wu D.Y., Zhang S.M., Xin Y., Chen Y., Yang S. (2003). PTEN encoding product: A marker for tumorigenesis and progression of gastric carcinoma. World J. Gastroenterol..

[72] Zheng H.-C., Qiu Y.-H., Zhao S. (2018). The roles of PTEN expression in gastric cancer: A bibliometric, meta and bioinformatics analysis. Oncotarget.

[73] Lee H.S., Lee H.K., Kim H.S., Yang H.K., Kim W.H. (2003). Tumour suppressor gene expression correlates with gastric cancer prognosis. J. Pathol..

[74] Sumiyoshi Y., Kakeji Y., Egashira A., Mizokami K., Orita H., Maehara Y. (2006). Overexpression of Hypoxia-Inducible Factor 1α and p53 Is a Marker for an Unfavorable Prognosis in Gastric Cancer. Clin. Cancer Res..

[75] Hammond E.M., Giaccia A.J. (2006). Hypoxia-Inducible Factor-1 and p53: Friends, Acquaintances, or Strangers?. Clin. Cancer Res..

[76] Sowter H.M., Ratcliffe P.J., Watson P., Greenberg A.H., Harris A.L. (2001). HIF-1-dependent regulation of hypoxic induction of the cell death factors BNIP3 and NIX in human tumors. Cancer Res..

[77] Kunz M., Ibrahim S., Koczan D., Thiesen H.J., Köhler H.J., Acker T., Plate K.H., Ludwig S., Rapp U.R., Bröcker E.B. (2001). Activation of c-Jun NH2-terminal kinase/stress-activated protein kinase (JNK/SAPK) is critical for hypoxia-induced apoptosis of human malignant melanoma. Cell Growth Differ. Mol. Biol. J. Am. Assoc. Cancer Res..

[78] Graeber T.G., Osmanian C., Jacks T., Housman D.E., Koch C.J., Lowe S.W., Giaccia A.J. (1996). Hypoxia-mediated selection of cells with diminished apoptotic potential in solid tumours. Nature.

[79] Wang Y., Pakunlu R.I., Tsao W., Pozharov V., Minko T. (2004). Bimodal Effect of Hypoxia in Cancer: Role of Hypoxia Inducible Factor in Apoptosis. Mol. Pharm..

[80] Dong Z., Venkatachalam M.A., Wang J., Patel Y., Saikumar P., Semenza G.L., Force T., Nishiyama J. (2001). Up-regulation of Apoptosis Inhibitory Protein IAP-2 by Hypoxia: HIF-1-independent mechanisms. J. Biol. Chem..

[81] Zhang L., Hill R.P. (2004). Hypoxia Enhances Metastatic Efficiency by Up-Regulating Mdm2 in KHT Cells and Increasing Resistance to Apoptosis. Cancer Res..

[82] Alvarez-Tejado M., Naranjo-Suárez S., Jiménez C., Carrera A.C., Landázuri M.O., del Peso L. (2001). Hypoxia Induces the Activation of the Phosphatidylinositol 3-Kinase/Akt Cell Survival Pathway in PC12 Cells: Protective Role in Apoptosis. J. Biol. Chem..

[83] Chae Y.C., Vaira V., Caino M.C., Tang H.-Y., Seo J.H., Kossenkov A.V., Ottobrini L., Martelli C., Lucignani G., Bertolini I. (2016). Mitochondrial Akt Regulation of Hypoxic Tumor Reprogramming. Cancer Cell.

[84] Kim M., Park S.Y., Pai H.S., Kim T.H., Billiar T.R., Seol D.W. (2004). Hypoxia Inhibits Tumor Necrosis Factor-Related Apoptosis-Inducing Ligand-Induced Apoptosis by Blocking Bax Translocation. Cancer Res..

[85] Greijer A.E., van der Wall E. (2004). The role of hypoxia inducible factor 1 (HIF-1) in hypoxia induced apoptosis. J. Clin. Pathol..

[86] Jia Y., Hu R., Li P., Zheng Y., Wang Y., Ma X. (2018). DEC1 is required for anti-apoptotic activity of gastric cancer cells under hypoxia by promoting Survivin expression. Gastric Cancer.

[87] Rohwer N., Welzel M., Daskalow K., Pfander D., Wiedenmann B., Detjen K., Cramer T. (2008). Hypoxia-Inducible Factor 1 Mediates Anoikis Resistance via Suppression of 5 Integrin. Cancer Res..

[88] Shay J.W., Wright W.E. (2000). Hayflick, his limit, and cellular ageing. Nat. Rev. Mol. Cell Biol..

[89] Heeg S. (2015). Variations in telomere maintenance and the role of telomerase inhibition in gastrointestinal cancer. Pharmacogenomics Pers. Med..

[90] Yuan X., Larsson C., Xu D. (2019). Mechanisms underlying the activation of TERT transcription and telomerase activity in human cancer: Old actors and new players. Oncogene.

[91] Seimiya H., Tanji M., Oh-Hara T., Tomida A., Naasani I., Tsuruo T. (1999). Hypoxia Up-Regulates Telomerase Activity via Mitogen-Activated Protein Kinase Signaling in Human Solid Tumor Cells. Biochem. Biophys. Res. Commun..

[92] Yatabe N., Kyo S., Maida Y., Nishi H., Nakamura M., Kanaya T., Tanaka M., Isaka K., Ogawa S., Inoue M. (2004). HIF-1-mediated activation of telomerase in cervical cancer cells. Oncogene.

[93] Lou F., Chen X., Jalink M., Zhu Q., Ge N., Zhao S., Fang X., Fan Y., Björkholm M., Liu Z. (2007). The Opposing Effect of Hypox-ia-Inducible Factor-2α on Expression of Telomerase Reverse Transcriptase. Mol. Cell. Biol..

[94] Wang Y.-P., Zhu Z.-Y., Tang Y., Ma Y.-C. (2017). Effects of acute hypoxia on telomere length of rat gastric mucosa tissue and underlying mechanism. Sheng Li Xue Bao [Acta Physiol. Sin.].

[95] Mahfouz N., Tahtouh R., Alaaeddine N., EL Hajj J., Sarkis R., Hachem R., Raad I., Hilal G. (2017). Gastrointestinal cancer cells treatment with bevacizumab activates a VEGF autoregulatory mechanism involving telomerase catalytic subunit hTERT via PI3K-AKT, HIF-1α and VEGF receptors. PLoS ONE.

[96] Sasaki T., Kuniyasu H., Luo Y., Kitayoshi M., Tanabe E., Kato D., Shinya S., Fujii K., Ohmori H., Yamashita Y. (2014). AKT Activation and Telomerase Reverse Transcriptase Expression are Concurrently Associated with Prognosis of Gastric Cancer. Pathobiology.

[97] Bae S.-K., Kim S.-R., Kim J.G., Kim J.Y., Koo T.H., Jang H.-O., Yun I., Yoo M.-A., Bae M.-K. (2006). Hypoxic induction of human visfatin gene is directly mediated by hypoxia-inducible factor-1. FEBS Lett..

[98] Segawa K., Fukuhara A., Hosogai N., Morita K., Okuno Y., Tanaka M., Nakagawa Y., Kihara S., Funahashi T., Komuro R. (2006). Visfatin in adipocytes is upregulated by hypoxia through HIF1α-dependent mechanism. Biochem. Biophys. Res. Commun..

[99] Mohammadi M., Zarghami N., Hedayati M., Ghaemmaghami S., Yamchi R.M., Mohaddes M. (2015). Visfatin effects on telomerase gene expression in AGS gastric cancer cell line. Indian J. Cancer.

[100] Lu G.-W., Wang Q.-J., Xia M.-M., Qian J. (2014). Elevated plasma visfatin levels correlate with poor prognosis of gastric cancer patients. Peptides.

[101] Krock B.L., Skuli N., Simon M.C. (2011). Hypoxia-Induced Angiogenesis: Good and Evil. Genes Cancer.

[102] Lv X., Li J., Zhang C., Hu T., Li S., He S., Yan H., Tan Y., Lei M., Wen M. (2017). The role of hypoxia-inducible factors in tumor angiogenesis and cell metabolism. Genes Dis..

[103] Urano N., Fujiwara Y., Doki Y., Tsujie M., Yamamoto H., Miyata H., Takiguchi S., Yasuda T., Yano M., Monden M. (2006). Overexpression of hypoxia-inducible factor-1 alpha in gastric adenocarcinoma. Gastric Cancer.

[104] Stoeltzing O., McCarty M.F., Wey J.S., Fan F., Liu W., Belcheva A., Bucana C.D., Semenza G.L., Ellis L.M. (2004). Role of Hypoxia-Inducible Factor 1α in Gastric Cancer Cell Growth, Angiogenesis, and Vessel Maturation. J. Natl. Cancer Inst..

[105] Huang S.-P., Wu M.-S., Shun C.-T., Wang H.-P., Hsieh C.-Y., Kuo M.-L., Lin J.-T. (2005). Cyclooxygenase-2 increases hypoxia-inducible factor-1 and vascular endothelial growth factor to promote angiogenesis in gastric carcinoma. J. Biomed. Sci..

[106] Meng F., Ding J., Liu N., Zhang J., Shao X., Shen H., Xue Y., Xie H., Fan D. (2005). Inhibition of gastric cancer angiogenesis by vector-based RNA interference for Raf-1. Cancer Biol. Ther..

[107] Van Beijnum J.R., Pieters W., Nowak-Sliwinska P., Griffioen A.W. (2017). Insulin-like growth factor axis targeting in cancer and tumour angiogenesis-The missing link. Biol. Rev..

[108] Li H., Adachi Y., Yamamoto H., Min Y., Ohashi H., Ii M., Arimura Y., Endo T., Lee C.-T., Carbone D.P. (2011). Insulin-like growth factor-I receptor blockade reduces tumor angiogenesis and enhances the effects of bevacizumab for a human gastric cancer cell line, MKN45. Cancer.

[109] Ru G.-Q., Zhao Z.-S., Tang Q.-L., Xu W.-J. (2007). Expressions of hypoxia inducible factor-1alpha and insulin-like growth factor-II in gastric carcinoma: Correlation with angiogenesis and prognosis. Zhonghua Wai Ke Za Zhi Chin. J. Surg..

[110] Na Seo A., Jung Y., Jang H., Lee E., Bae H.-I., Son T., Kwon O., Chung H.Y., Yu W., Lee Y.M. (2019). Clinical significance and prognostic role of hypoxia-induced microRNA 382 in gastric adenocarcinoma. PLoS ONE.

[111] Lamouille S., Xu J., Derynck R. (2014). Molecular mechanisms of epithelial–mesenchymal transition. Nat. Rev. Mol. Cell Biol..

[112] Roche J. (2018). The Epithelial-to-Mesenchymal Transition in Cancer. Cancers.

[113] Lambert A.W., Pattabiraman D.R., Weinberg R.A. (2017). Emerging Biological Principles of Metastasis. Cell.

[114] Jiang J., Tang Y.-L., Liang X.-H. (2011). EMT: A new vision of hypoxia promoting cancer progression. Cancer Biol. Ther..

[115] Peng Z., Wang C.X., Fang E.H., Wang G.B., Tong Q. (2014). Role of epithelial-mesenchymal transition in gastric cancer initiation and progression. World J. Gastroenterol..

[116] Kalluri R., Weinberg R.A. (2009). The basics of epithelial-mesenchymal transition. J. Clin. Investig..

[117] Furuta C., Miyamoto T., Takagi T., Noguchi Y., Kaneko J., Itoh S., Watanabe T., Itoh F. (2015). Transforming growth factor-β signaling enhancement by long-term exposure to hypoxia in a tumor microenvironment composed of Lewis lung carcinoma cells. Cancer Sci..

[118] Wendt M.K., Allington T.M., Schiemann W.P. (2009). Mechanisms of the epithelial–mesenchymal transition by TGF-β. Future Oncol..

[119] Bakin A., Rinehart C., Tomlinson A.K., Arteaga C.L. (2002). p38 mitogen-activated protein kinase is required for TGFβ-mediated fibroblastic transdifferentiation and cell migration. J. Cell Sci..

[120] Lamouille S., Derynck R. (2007). Cell size and invasion in TGF-β–induced epithelial to mesenchymal transition is regulated by activation of the mTOR pathway. J. Cell Biol..

[121] Azuma M., Motegi K., Aota K., Yamashita T., Yoshida H., Sato M. (1999). TGF-β1 Inhibits NF-κB Activity through Induction of IκB-α Expression in Human Salivary Gland Cells: A Possible Mechanism of Growth Suppression by TGF-β1. Exp. Cell Res..

[122] Jiang Y.G., Luo Y., He D.L., Li X., Zhang L.L., Peng T., Li M.C., Lin Y.H. (2007). Role of Wnt/β-catenin signaling pathway in epithelial-mesenchymal transition of human prostate cancer induced by hypoxia-inducible factor-1α. Int. J. Urol..

[123] Sahlgren C., Gustafsson M.V., Jin S., Poellinger L., Lendahl U. (2008). Notch signaling mediates hypoxia-induced tumor cell migration and invasion. Proc. Natl. Acad. Sci USA.

[124] Chen J., Imanaka N., Chen J., Griffin J.D. (2010). Hypoxia potentiates Notch signaling in breast cancer leading to decreased E-cadherin expression and increased cell migration and invasion. Br. J. Cancer.

[125] Lee J., Cristescu R., Kim K.-M., Kim K., Kim S.T., Park S.H., Kang W.K. (2017). Development of mesenchymal subtype gene signature for clinical application in gastric cancer. Oncotarget.

[126] Susman S., Barnoud R., Bibeau F., Borrini F., Pocard M., Tomuleasa C., Sabourin J.C. (2015). The Lauren Classification Highlights the Role of Epithelialto- Mesenchymal Transition in Gastric Carcinogenesis: An Immunohistochemistry Study of the STAT3 and Adhesion Molecules Expression. J. Gastrointest. Liver Dis..

[127] Kato Y., Yashiro M., Noda S., Tendo M., Kashiwagi S., Doi Y., Nishii T., Matsuoka J., Fuyuhiro Y., Shinto O. (2010). Establishment and characterization of a new hypoxia-resistant cancer cell line, OCUM-12/Hypo, derived from a scirrhous gastric carcinoma. Br. J. Cancer.

[128] Matsuoka J., Yashiro M., Doi Y., Fuyuhiro Y., Kato Y., Shinto O., Noda S., Kashiwagi S., Aomatsu N., Hirakawa T. (2013). Hypoxia Stimulates the EMT of Gastric Cancer Cells through Autocrine TGFβ Signaling. PLoS ONE.

[129] Noda S., Yashiro M., Nshii T., Hirakawa K. (2010). Hypoxia upregulates adhesion ability to peritoneum through a transforming growth factor-β-dependent mechanism in diffuse-type gastric cancer cells. Eur. J. Cancer.

[130] Liu L., Sun L., Zhao P., Yao L., Jin H., Liang S., Wang Y., Zhang D., Pang Y., Shi Y. (2010). Hypoxia promotes metastasis in human gastric cancer by up-regulating the 67-kDa laminin receptor. Cancer Sci..

[131] Oh Y.S., Kim H.Y., Song I.-C., Yun H.-J., Jo D.-Y., Kim S., Lee H.J. (2012). Hypoxia induces CXCR4 expression and biological activity in gastric cancer cells through activation of hypoxia-inducible factor-1α. Oncol. Rep..

[132] Xia X., Wang S., Ni B., Xing S., Cao H., Zhang Z., Yu F., Zhao E., Zhao G. (2020). Hypoxic gastric cancer-derived exosomes promote progression and metastasis via MiR-301a-3p/PHD3/HIF-1α positive feedback loop. Oncogene.

[133] Reynolds T.Y., Rockwell S., Glazer P. (1996). Genetic instability induced by the tumor microenvironment. Cancer Res..

[134] Bindra R.S., Glazer P.M. (2007). Co-repression of mismatch repair gene expression by hypoxia in cancer cells: Role of the Myc/Max network. Cancer Lett..

[135] Rodríguez-Jiménez F.J., Moreno-Manzano V., Lucas-Dominguez R., Sánchez-Puelles J.M. (2008). Hypoxia Causes Downregulation of Mismatch Repair System and Genomic Instability in Stem Cells. Stem Cells.

[136] Yuan J., Narayanan L., Rockwell S., Glazer P.M. (2000). Diminished DNA repair and elevated mutagenesis in mammalian cells exposed to hypoxia and low pH. Cancer Res..

[137] Bindra R.S., Glazer P.M. (2005). Genetic instability and the tumor microenvironment: Towards the concept of microenvironment-induced mutagenesis. Mutat. Res. Mol. Mech. Mutagen..

[138] Koi M., Boland C.R. (2011). Tumor hypoxia and genetic alterations in sporadic cancers. J. Obstet. Gynaecol. Res..

[139] Bindra R.S., Schaffer P.J., Meng A., Woo J., Måseide K., Roth M.E., Lizardi P., Hedley D.W., Bristow R.G., Glazer P.M. (2004). Down-Regulation of Rad51 and Decreased Homologous Recombination in Hypoxic Cancer Cells. Mol. Cell. Biol..

[140] Rofstad E.K., Johnsen N.M., Lyng H. (1996). Hypoxia-induced tetraploidisation of a diploid human melanoma cell line in vitro. Br. J. Cancer Suppl..

[141] Lopez-Sánchez L.M., Jimenez C., Valverde A., Hernández V., Peñarando J., Martinez A., López-Pedrera C., Muñoz-Castañeda J.R., De la Haba-Rodríguez J.R., Aranda E. (2014). CoCl_2_, a Mimic of Hypoxia, Induces Formation of Polyploid Giant Cells with Stem Characteristics in Colon Cancer. PLoS ONE.

[142] Bhandari V., Li C.H., Bristow R.G., Boutros P.C., PCAWG Consortium (2020). Divergent mutational processes distinguish hypoxic and normoxic tumours. Nat. Commun..

[143] Hudler P. (2012). Genetic Aspects of Gastric Cancer Instability. Sci. World J..

[144] Vander Heiden M.G., Cantley L.C., Thompson C.B. (2009). Understanding the Warburg Effect: The Metabolic Requirements of Cell Proliferation. Science.

[145] Ghanbari Movahed Z.G., Rastegari-Pouyani M., Mohammadi M., Mansouri K. (2019). Cancer cells change their glucose metabolism to overcome increased ROS: One step from cancer cell to cancer stem cell?. Biomed. Pharmacother..

[146] DeBerardinis R.J., Mancuso A., Daikhin E., Nissim I., Yudkoff M., Wehrli S., Thompson C.B. (2007). Beyond aerobic glycolysis: Transformed cells can engage in glutamine metabolism that exceeds the requirement for protein and nucleotide synthesis. Proc. Natl. Acad. Sci. USA.

[147] Liberti M.V., Locasale J.W. (2016). The Warburg Effect: How Does it Benefit Cancer Cells?. Trends Biochem. Sci..

[148] Semenza G.L. (2010). HIF-1: Upstream and downstream of cancer metabolism. Curr. Opin. Genet. Dev..

[149] Airley R.E., Mobasheri A. (2007). Hypoxic Regulation of Glucose Transport, Anaerobic Metabolism and Angiogenesis in Cancer: Novel Pathways and Targets for Anticancer Therapeutics. Chemotherapy.

[150] Semenza G.L., Jiang B.-H., Leung S.W., Passantino R., Concordet J.-P., Maire P., Giallongo A. (1996). Hypoxia Response Elements in the Aldolase A, Enolase 1, and Lactate Dehydrogenase A Gene Promoters Contain Essential Binding Sites for Hypoxia-inducible Factor 1. J. Biol. Chem..

[151] Kim J.-W., Tchernyshyov I., Semenza G.L., Dang C.V. (2006). HIF-1-mediated expression of pyruvate dehydrogenase kinase: A metabolic switch required for cellular adaptation to hypoxia. Cell Metab..

[152] Song I.-S., Wang A.-G., Yoon S.Y., Kim J.-M., Kim J.H., Lee D.-S., Kim N.-S. (2009). Regulation of glucose metabolism-related genes and VEGF by HIF-1α and HIF-1β, but not HIF-2α, in gastric cancer. Exp. Mol. Med..

[153] Rho M., Kim J., Jee C.D., Lee Y.M., Lee H.E., Kim M.A., Lee H.S., Kim W.H. (2007). Expression of type 2 hexokinase and mitochondria-related genes in gastric carcinoma tissues and cell lines. Anticancer Res..

[154] Xu G., Li M., Wu J., Qin C., Tao Y., He H. (2020). Circular RNA circNRIP1 Sponges microRNA-138-5p to Maintain Hypoxia-Induced Resistance to 5-Fluorouracil Through HIF-1α-Dependent Glucose Metabolism in Gastric Carcinoma. Cancer Manag. Res..

[155] Noman M.Z., Hasmim M., Messai Y., Terry S., Kieda C., Janji B., Chouaib S. (2015). Hypoxia: A key player in antitumor immune response. A Review in the Theme: Cellular Responses to Hypoxia. Am. J. Physiol. Cell Physiol..

[156] Zhou J., Tang Z., Gao S., Li C., Feng Y., Zhou X. (2020). Tumor-Associated Macrophages: Recent Insights and Therapies. Front. Oncol..

[157] Lewis C., Murdoch C. (2005). Macrophage Responses to Hypoxia: Implications for Tumor Progression and Anti-Cancer Therapies. Am. J. Pathol..

[158] Tao L.-L., Shi S.-J., Chen L.-B., Huang G.-C. (2014). Expression of monocyte chemotactic protein-1/CCL2 in gastric cancer and its relationship with tumor hypoxia. World J. Gastroenterol..

[159] Osinsky S., Bubnovskaya L., Ganusevich I., Kovelskaya A., Gumenyuk L., Olijnichenko G., Merentsev S. (2011). Hypoxia, tumour-associated macrophages, microvessel density, VEGF and matrix metalloproteinases in human gastric cancer: Interaction and impact on survival. Clin. Transl. Oncol..

[160] Zhang W.J., Chen C., Zhou Z.H., Gao S.T., Tee T.J., Yang L.Q., Xu Y.Y., Pang T.H., Xu X.Y., Sun Q. (2017). Hypoxia-inducible factor-1 alpha Correlates with Tumor-Associated Macrophages Infiltration, Influences Survival of Gastric Cancer Patients. J. Cancer.

[161] Shen Z., Kauttu T., Seppänen H., Vainionpää S., Ye Y., Wang S., Mustonen H., Puolakkainen P. (2013). Both macrophages and hypoxia play critical role in regulating invasion of gastric cancer in vitro. Acta Oncol..

[162] Zhihua Y., Yulin T., Yibo W., Wei D., Yin C., Jiahao X., Runqiu J., Xuezhong X. (2019). Hypoxia decreases macrophage glycolysis and M1 percentage by targeting microRNA-30c and mTOR in human gastric cancer. Cancer Sci..

[163] Ping W., Senyan H., Li G., Yan C., Long L. (2018). Increased Lactate in Gastric Cancer Tumor-Infiltrating Lymphocytes Is Related to Impaired T Cell Function Due to miR-34a Deregulated Lactate Dehydrogenase A. Cell. Physiol. Biochem..

[164] Ohue Y., Nishikawa H. (2019). Regulatory T (Treg) cells in cancer: Can Treg cells be a new therapeutic target?. Cancer Sci..

[165] Deng B., Zhu J.M., Wang Y., Liu T.T., Ding Y.B., Xiao W.M., Lu G.-T., Bo P., Shen X.Z. (2013). Intratumor Hypoxia Promotes Immune Tolerance by Inducing Regulatory T Cells via TGF-β1 in Gastric Cancer. PLoS ONE.

[166] Grivennikov S.I., Greten F.R., Karin M. (2010). Immunity, inflammation, and cancer. Cell.

[167] Baker K.J., Houston A., Brint E. (2019). IL-1 Family Members in Cancer; Two Sides to Every Story. Front. Immunol..

[168] Xuan Y., Wang Y.N. (2017). Hypoxia/IL-1α axis promotes gastric cancer progression and drug resistance. J. Dig. Dis..

[169] Nishikawa J., Iizasa H., Yoshiyama H., Shimokuri K., Kobayashi Y., Sasaki S., Nakamura M., Yanai H., Sakai K., Suehiro Y. (2018). Clinical Importance of Epstein–Barr Virus-Associated Gastric Cancer. Cancers.

[170] Zhang X.Y., Zhang P.Y., Aboul-Soud M.A. (2017). From inflammation to gastric cancer: Role of Helicobacter pylori. Oncol. Lett..

[171] Bass A.J., Thorsson V., Shmulevich I., Reynolds S.M., Miller M., Bernard B., Hinoue T., Laird P.W., Curtis C., Shen H. (2014). Comprehensive molecular characterization of gastric adenocarcinoma. Nature.

[172] Zhu C., Zhu Q., Wang C., Zhang L., Wei F., Cai Q. (2016). Hostile takeover: Manipulation of HIF-1 signaling in pathogen-associated cancers (Review). Int. J. Oncol..

[173] Risna W., GontarAlamsyah S., Dharma L. (2020). Comparison of hypoxic inducible factor 1 alpha (hif1α) between gastritis positive and negative helicobacter pylori. Int. J. Res. Sci. Manag..

[174] Park J.-H., Kim T.-Y., Jong H.-S., Kim T.Y., Chun Y.-S., Park J.-W., Lee C.-T., Jung H.C., Kim N.K., Bang Y.-J. (2003). Gastric Epithelial Reactive Oxygen Species Prevent Normoxic Degradation of Hypoxia-inducible Factor-1α in Gastric Cancer Cells. Clin. Cancer Res..

[175] Lee D.Y., Jung D.E., Yu S.S., Lee Y.S., Choi B.K., Lee Y.C. (2017). Regulation of SIRT3 signal related metabolic reprogramming in gastric cancer by *Helicobacter pylori* oncoprotein CagA. Oncotarget.

[176] Valenzuela-Valderrama M., Cerda-Opazo P., Backert S., González M.F., Carrasco-Véliz N., Jorquera-Cordero C., Wehinger S., Canales J., Bravo D., Quest A.F.G. (2019). The Helicobacter pylori Urease Virulence Factor Is Required for the Induction of Hypoxia-Induced Factor-1α in Gastric Cells. Cancers.

[177] Bhattacharyya A., Chattopadhyay R., Hall E.H., Mebrahtu S.T., Ernst P.B., Crowe S.E. (2010). Mechanism of hypoxia-inducible factor 1α-mediated Mcl1 regulation in Helicobacter pylori-infected human gastric epithelium. Am. J. Physiol. Liver Physiol..

[178] Matak P., Heinis M., Mathieu J.R.R., Corriden R., Cuvellier S., Delga S., Mounier R., Rouquette A., Raymond J., Lamarque D. (2015). Myeloid HIF-1 Is Protective in *Helicobacter pylori*–Mediated Gastritis. J. Immunol..

[179] Kraus R.J., Yu X., Cordes B.-L.A., Sathiamoorthi S., Iempridee T., Nawandar D.M., Ma S., Romero-Masters J.C., McChesney K.G., Lin Z. (2017). Hypoxia-inducible factor-1α plays roles in Epstein-Barr virus’s natural life cycle and tumorigenesis by inducing lytic infection through direct binding to the immediate-early BZLF1 gene promoter. PLOS Pathog..

[180] Xiang T., Lin Y.-X., Ma W., Zhang H.-J., Chen K.-M., He G.-P., Zhang X., Xu M., Feng Q.-S., Chen M.-Y. (2018). Vasculogenic mimicry formation in EBV-associated epithelial malignancies. Nat. Commun..

[181] Holohan C., Van Schaeybroeck S., Longley D.B., Johnston P.G. (2013). Cancer drug resistance: An evolving paradigm. Nat. Rev. Cancer.

[182] Ulivi P., Marisi G., Passardi A. (2016). Relationship between hypoxia and response to antiangiogenic therapy in metastatic colorectal cancer. Oncotarget.

